# Angiotensin II promotes ovarian cancer spheroid formation and metastasis by upregulation of lipid desaturation and suppression of endoplasmic reticulum stress

**DOI:** 10.1186/s13046-019-1127-x

**Published:** 2019-03-07

**Authors:** Qingyu Zhang, Shan Yu, Melody Man Ting Lam, Terence Chuen Wai Poon, Litao Sun, Yufei Jiao, Alice Sze Tsai Wong, Leo Tsz On Lee

**Affiliations:** 1 Centre of Reproduction, Development and Aging, Faculty of Health Sciences, University of Macau, Taipa, Macau; 2 Proteomics, Metabolomics and Drug Development Core, Faculty of Health Sciences, University of Macau, Taipa, Macau; 3 Department of Ultrasound, The Secondary Affiliated Hospital of Harbin Medical University, Harbin, China; 4 Department of Pathology, The Secondary Affiliated Hospital of Harbin Medical University, Harbin, China; 5 School of Biological Sciences, The University of Hong Kong, Pokfulam Road, Hong Kong

**Keywords:** Angiotensin II, Ovarian cancer, Spheroid formation, Lipogenesis, Endoplasmic reticulum stress

## Abstract

**Background:**

Angiotensin II (ANGII) and its receptor (AGTR1) have been proposed as significant contributors to metastasis in multiple cancers. Further, high AGTR1 levels are associated with poor epithelial ovarian cancer (EOC) outcomes. However, the mechanistic basis for these effects is unknown. Recent studies have suggested that ovarian cancer metastasis is highly dependent on the formation of multicellular spheroids (MCS). To understand the associations between the ANGII/AGTR1 pathway and cancer outcomes, we evaluated the effects of ANGII on MCS formation by ovarian cancer cells and used a proteomic approach to analyze the mechanistic basis.

**Methods:**

We used the data from the GENT database and immunohistochemistry staining to assess the AGTR1 expression in epithelial ovarian cancer (EOC) patients and to assess its role in cancer progression. Colony formation assay, 3D culture assay, and transwell assays were used to analyze the effect of ANGII on the MCS formation and cell migration. The signaling pathways of AGTR1 and transactivation of epidermal growth factor receptor (EGFR) transactivation were investigated by the western blotting analysis. Xenograft models were used to determine the role of AGTR1 in ovarian cancer metastasis. ANGII release from ovarian cancer cells and ANGII levels in the EOC ascites fluid were measured by immunoassay. A shotgun proteomic approach was used to explore the detail molecular mechanism. Modulation of lipid desaturation and endoplasmic reticulum stress were verified by the in vitro and in vivo functional assays.

**Results:**

AGTR1 expression was negatively correlated with EOC prognosis. AGTR1activation significantly enhanced the MCS formation and cell migration. ANGII triggered both of the classical AGTR1 pathway and the EGFR transactivation. ANGII administration increased peritoneal metastasis. In addition, ovarian cancer cells secreted ANGII and enhanced cancer metastasis in a positive feedback manner. Based on the proteomic data, lipid desaturation was activated by induction of stearoyl-CoA desaturase-1 (SCD1), which suggests that inhibition of SCD1 may significantly reduce MCS formation by increasing endoplasmic reticulum stress.

**Conclusions:**

ANGII promotes MCS formation and peritoneal metastasis of EOC cells. AGTR1 activation increases the lipid desaturation via SCD1 upregulation, which ultimately reduces endoplasmic reticulum stress in MCS. This mechanism explained the association between high levels of AGTR1 and poor clinical outcomes in EOC patients.

**Electronic supplementary material:**

The online version of this article (10.1186/s13046-019-1127-x) contains supplementary material, which is available to authorized users.

## Background

Ovarian cancer is the second most common and the most lethal gynecologic cancer [1]. This lethality is due to late diagnosis of the disease, with > 70% of patients diagnosed with an advanced/metastatic stage (stage III and IV). The disease progresses aggressively throughout the peritoneal cavity in an asymptomatic manner [2]. Unlike most solid tumors, ovarian cancer rarely metastasizes via the blood, but rather disseminates throughout the peritoneal cavity. Dissemination is highly aggressive, in a feed-forward manner, posing a unique treatment challenge for advanced/metastatic ovarian cancer. Ovarian carcinoma metastasizes to other neighboring organs by direct contact, for example to the bladder or colon [3]. Cancer cells also detach from the primary ovarian tumor and spread throughout the peritoneum, affecting multiple organs.

Since ovarian cancer can metastasize through the peritoneal fluid, metastasis is markedly different than the hematogenous route of other cancers. Recent findings have demonstrated metastasis to be linked to epithelial-to-mesenchymal transition (EMT), the formation of multicellular spheroids (MCS), and the development of stem cell properties [4]. In this process, detached tumor cells aggregate as MCS within the abdominal cavity to overcome anoikis. These spheroids spread throughout the peritoneal cavity, invade the peritoneum, and implant in pelvic organs and the omentum. Moreover, MCS in the ascites of ovarian cancer patients is a major impediment to effective treatment and correlate with poor clinical outcomes.

Recent evidence has demonstrated that angiotensin II type I receptor (AGTR1), is involved in tumor progression and metastasis. AGTR1 is a well-known receptor that regulates the cardiovascular system. In fact, AGTR1 is proposed to be involved in several types of gynecological malignancies including endometrial cancer [5] and cervical carcinoma [6]. Expression of AGTR1 has been found in several human malignant tumors, including breast [7], skin [8], and prostate [9], as well as gynecologic cancers [10, 11]. Further, angiotensin II (ANGII) and AGTR1 play essential roles in tumor survival, angiogenesis, and metastasis. For example, an antagonist of AGTR1 inhibits the migration and invasion of human lung adenocarcinoma cells through inactivation of the PI3K/AKT and MAPK signaling pathways [12]. ANGII increases metalloproteinase-2 (MMP2) and MMP14 activities via the MAPK pathway [13]. In vitro, ANGII stimulates cellular proliferation and vascular endothelial growth factor (VEGF) secretion by gynecologic cancer cells [14].

In ovarian cancer, the frequency of AGTR1-positive cells is very high, with 85% of invasive adenocarcinomas expressing AGTR1 [15]. This finding suggests that ANGII and AGTR1 may play crucial roles in the biology of ovarian cancer and could be promising therapeutic targets. A recent study has demonstrated serum angiotensin-converting enzyme (ACE) levels to be significantly higher in epithelial ovarian cancer (EOC) patients than in a control group [16]. Further, the expression of AGTR1 can be induced by BRCA1 [17]. In summary, current data indicate that the study of AGTR1 pathways may reveal new avenues for the investigation of ovarian cancer pathogenesis. However, the molecular basis for how the ANGII/AGTR1 axis influences ovarian cancer is unclear. Herein, a role for ANGII and AGTR1 in ovarian cancer spheroid formation is identified. A clearer understanding of the molecular mechanistic basis for ovarian cancer will provide for development of new and efficacious therapies for this deadly malignancy. Moreover, a role for ANGII has been suggested in other cancers and the data herein may provide insight into the role of ANGII in other cancers, with regard to MCS formation.

The data herein suggest ANGII treatment to significantly increase the spheroid formation, growth and the invasiveness of multiple ovarian cancer cell lines. These activities are mediated by classic direct activation of the MAPK/ERK pathway and transactivation of the epidermal growth factor receptor (EGFR). Interestingly, a dramatic increase in ANGII production and release are found in ovarian cancer cells. This suggests that ANGII forms a positive feedback loop that enhances cancer progression and metastasis. Matched with our hypothesis, the ANGII levels in ascites of ovarian cancer cell patients are significantly higher than in non-cancerous patients. In a xenograft model, intraperitoneal (i.p.) injection of ANGII significantly increases the tumorigenicity and metastasis of ovarian cancer cells, whereas an AGTR1 antagonist, losartan, suppresses this effect. Moreover, a proteomic approach was used to describe the molecular ANGII/AGTR1 axis in ovarian cancer and those data suggest that ANGII regulates lipid homeostasis. Of the highly upregulated genes, we focused on Stearoyl-CoA Desaturase (SCD1), which is involved in the actions of ANGII in terms of spheroid formation, endoplasmic reticulum (ER) stress, as well as tumorigenicity and metastasis in the xenograft model. Hence, our data suggest that the positive feedback loop of ANGII is one of the major pathways that promotes ovarian cancer development, by enhancing spheroid formation within the peritoneal cavity. The increase in spheroid formation is mediated by upregulation of SCD1 gene expression, affecting lipid homeostasis and suppressing ER stress within the spheroid.

## Materials and methods

### Cell lines and cell culture

The cancer cell line A2780 was obtained from the American Type Culture Collection (ATCC). The Ovca429 cell line was kindly provided by Prof. S.W. Taso of The University of Hong Kong. The isogenic highly metastatic (HM) and non-metastatic (NM) cells were generated from SKOV3.ip1 as described previously [18]. For the transwell assay, migrated cells on the lower surface of the filter and non-migrated cells were separately harvested, and digested into single-cell suspensions. Isolated single-cell clones were selected and verified by several in vitro and in vivo assays, including the spheroid formation assay, xenograft experiments, and spectral karyotyping. The HM cells exhibited a strong metastatic signature, unlike the NM cells, which failed to form detectable metastases. Therefore, this HM/NM model offers a well-controlled experimental system for studying the metastasis of ovarian cancer. Ovca429, A2780, and HM were maintained in Dulbecco’s Modified Eagle’s Medium (DMEM) supplemented with 10% fetal bovine serum (FBS) and 100 U/ml of penicillin and 100 μg/ml of streptomycin (Gibco) at 37 °C with 5% CO_2_.

### Human samples

The research protocol was approved by the Harbin Medical University Secondary Affiliated Hospital research ethics committee. Patients diagnosed with ovarian carcinoma between December 2016 and April 2018 were included in this study. The tumor samples were fixed with formalin. Informed consents were obtained prior to the following experimental and clinical data analysis and integration.

### Quantitative real-time polymerase chain reaction (RT-qPCR)

Total RNA was isolated using TriPure Isolation Reagent (Roche). Total RNA (1 μg) was reverse-transcribed with an oligo-dT primer and Superscript III reverse transcriptase (Invitrogen). RT-qPCR analyses of target genes were performed by the SYBR Green PCR Master Mix (Applied Biosystems) with gene specific primers (Additional file 1: Table S1). The fluorescence signals were measured with an ABI 7300 Real-Time PCR System (Applied Biosystems). The ratio change in the target gene relative to the GAPDH house keeping gene control was determined by the 2^-ΔΔCt^ method [19].

### Spheroid formation assay

Agarose (0.5%) was pre-coated on 100 mm culture plates (Corning). To generate spheroids, ovarian cancer cells (50000) (Ovca429, A2780, or HM) were seeded on the pre-coated plates and cultured in medium supplemented with 10% FBS for 8–10 days. Spheroids were collected by centrifugation and re-seeded in 6-well plates and incubated for 12 h for cell attachment. After attachment, unattached multicellular aggregates were removed. The adhered MCS were stained with 0.5% crystal violet and the number of spheroids calculated by ImageJ software.

### 3D culture assay

Agarose (0.5%, 30 μL per well) was used to pre-coat 96-well plates. Ovarian cancer cells were seeded to the plates together with Matrigel (500 cells in 100 μL culture medium with 100 μL Matrigel; BD). For ANGII and losartan treatments, a 2 x drug concentration was added directly into the culture medium. After 30–60 min matrix gel polymerization, another 100 μL of medium containing a 1 x concentration of the corresponding drug was added. The plates were then incubated at 37 °C at 5% without disturbance for 10 days. The growth of MCS was observed by light microscopy and the size of the MCS (diameter) calculated. To measure the cell proliferation of the spheroids, they were reseeded to culture plates and incubated for 2 days. The cells were then stained with 0.5% crystal violet and growth area assessed.

### Western blotting

Briefly, cells were collected and lysed with radioimmunoprecipitation assay buffer (RIPA) (50 mM Tris pH 7.4, 0.25% Na-deoxycholate, 1% NP-40, 150 mM NaCl and 1 mM EDTA) with proteinase and phosphatase inhibitors (Roche). Protein concentration was measured by Coomassie® Brilliant Blue (Bio-Rad). 20–30 μg total protein was separated by 8–10% sodium dodecyl sulfate polyacrylamide gel electrophoresis (SDS-PAGE) and transferred to polyvinyl difluoride (PVDF) membranes (Bio-Rad) with a Trans-Blot® Turbo™ Transfer System. For membrane blotting, 5% non-fat dry milk in Tris-buffered saline with Tween 20 (TBST) was used. After incubation with primary antibodies in TBST (The details of the antibodies and the dilutions are listed in Additional file 1: Table S2), the membranes were subsequently incubated with horseradish peroxidase (HRP)-conjugated secondary antibody (1:3000 dilution, Bio-Rad). The signals were detected by the Clarity Western ECL substrate (Bio-Rad) and captured by ChemiDoc™ XRS+ Imaging Systems (Bio-Rad). Protein relative levels were quantified by analyzing the ratio of band intensity of target protein versus loading control using ImageJ.

### Immunohistochemistry

Formalin-fixed tissue sections were prepared as described previously [20]. Deparaffinized tissue sections were stained with AGTR1 antibody after epitope retrieval by microwave. Immunohistochemistry (IHC) was performed by a standard automated IHC procedure (Dako Autostainer Universal System). The primary antibody for AGTR1 (rabbit polyclonal, MBS244122) was used at a dilution of 1:200, and negative controls were treated with PBS. The secondary antibody was anti-rabbit-HRP conjugated and used at a dilution of 1:500. The investigator conducting IHC (Yu Shan) was blinded to patient clinical data. The grading criteria were as follows: location of signal (cytoplasm negative 0, cytoplasm 1, mixed 2, and membrane 3); signaling strength (negative 0, weak 1, moderate 2, and strong 3). IHC scores were calculated as the sum of location scores and signaling strength scores.

### Construction of constitutive active mutant (CAM) of AGTR1 stable expression cells

A site-directed mutagenesis kit (NEB, E0554s) was used to introduce point mutations (AGTR1-N111S and AGTR1-L305Q) into the pEGFP-N1-AGTR1 plasmid. The mutations were confirmed by Sanger sequencing. The mutated plasmids were transfected into Ovca429 cells and selected with G418 for one month to establish stable cell lines. The surviving cells after G418 selection were further selected from a single colony and validated by PCR identification. AGTR1 modified cells were used for spheroid formation and xenograft experiments.

### Xenograft model of ovarian cancer

NOD-SCID 6–8 week-old females (Charles River Laboratories, Wilmington, MA) were used for tumor xenograft experiments. All animal studies were performed according to the guidelines and regulations set by the Animal Research Ethics Committee of University of Macau (UMARE-029-2017). Ovarian cancer cells (Ovca429), 4 × 10^6^, were suspended in 0.2 mL Hanks’ balanced salt solution and were injected into the peritoneal cavity. The day after inoculation, the mice were randomly separated into groups and treated with different drugs dissolved in Hanks’ buffer. After one month, the mice were sacrificed and the number and weight of disseminated tumor nodules within the peritoneal cavity measured.

### Apoptosis/necrosis assay

The cells were harvested after drug treatment and resuspended in Annexin V-binding buffer at 1 × 10^6^ cells/mL. For each 100 μL sample, 5 μL of the Annexin V FITC conjugate was added and incubated at room temperature for 15 min. Next, 5 μL of Propidium Iodide (PI, 1.0 mg/mL) was added for 15 min for the staining of dead cells. After the incubation, 400 μL of Annexin V-binding buffer was added with samples kept on ice until analysis. The stained cells were analyzed by flow cytometry (BD Accuri™ C6 Flow Cytometer, with at least 10,000 events).

### Liquid chromatography (LC)-tandem mass spectrometry (MS/MS) analysis

Cells were treated with ANGII in 6-well plates with 200 μL of lysis buffer (0 .1M Tris-HCL, pH 7.5, 4% SDS, 0 .1M DTT) used for each well. The cells were collected with a cell scraper and vortexed 3 times for 5 min each. The lysates were sonicated with pulses of 30s on and 30s off, 20 times, at 4 °C and then incubated at 56 °C for 30 min. After centrifugation (16,000 x g for 5 min) supernatants were collected for digestion. The protein amount was measured using the Bio-Rad RC-DC protein assay, as instructed by the product manual. (Bio-Rad RC DC™ Protein Assay Kit II, 5000122). Total protein (12.5 μg) was digested with trypsin (trypsin, enzyme to protein ratio 1:20) and vortexed for 1 min and incubated at 37 °C for 16 h. After drying the peptide extract completely with a Speed Vac, 15 μL of 5% acetonitrile/0.1% formic acid was used to reconstitute the samples, which were then sonicated for 5 min. All contents were transferred to a new HPLC vial-Thermo #MSCERT5000-36LVW. A total of 6 μL was injected for LC-MS analysis [21]. Label-free quantification was obtained by MaxQuant 1.5.3.17. A database search was performed using PEAKS Studio 8.5.

### RNA interference

In this study, RNA interference was used to silence target genes (AGTR1, SCD1). The silencing of AGTR1 was accomplished by transfecting siRNAs against AGTR1 into cancer cells. siRNA smart pool (L-005428-00-0005) from Dharmacon for AGTR1 interference was used for transfection. During transfection 20–40 pmol/well (6-well plates) of siRNA were transfected into the target cells with lipofectamine 3000. For the knock-down of SCD1, we used short hairpin RNA (shRNA) silencing plasmids. The SCD1-shRNA plasmids were purchased from Genepharma (Shanghai, China). SCD1-shRNA was constructed into a pGPU6/GFP/Neo vector. The sequences were as follows: shSCD1–1#: GAGATAAGTTGGAGACGATGC, shSCD1–2#: GGTACTACAAACCTGGCTTGC; shSCD1–3#: GCGATATGCTGTGGTGCTTAA; shSCD1–4#: GCACATCAACTTCACCACATT; the non-targeting shRNA (shNC): TTC TCC GAA CGT GTC ACG T. Similar to the siRNA, the shRNA plasmids were transfected into the target cells in 6-well plates (2 μg / well) with lipofectamine 3000. After 24 h, the cells were harvested for real-time PCR analysis, western blotting or functional assay.

### Bioinformatics analysis

We compared AGTR1 expression in normal ovary tissues and in ovarian cancer tissue by searching gene symbol “AGTR1” and source type of “tissues” (*http://medicalgenome.kribb.re.kr/GENT/*) [22]. The gene expression profiles of ovarian cancer and normal ovary were extracted and presented as scatter plots. The outlier analysis tool in the Oncomine Platform was employed to identify the signature genes that were significantly upregulated in independent studies of ovarian cancer (*https://www.oncomine.org/resource/login*) [23, 24]. The AGTR1 gene expression correlation with prognosis was analyzed by using a Kaplan Meier (KM) plotter (*http://kmplot.com/analysis/*) [25].

## Results

### High expression of AGTR1 is negatively correlated with prognosis in ovarian cancer patients

**Fig. 1:**
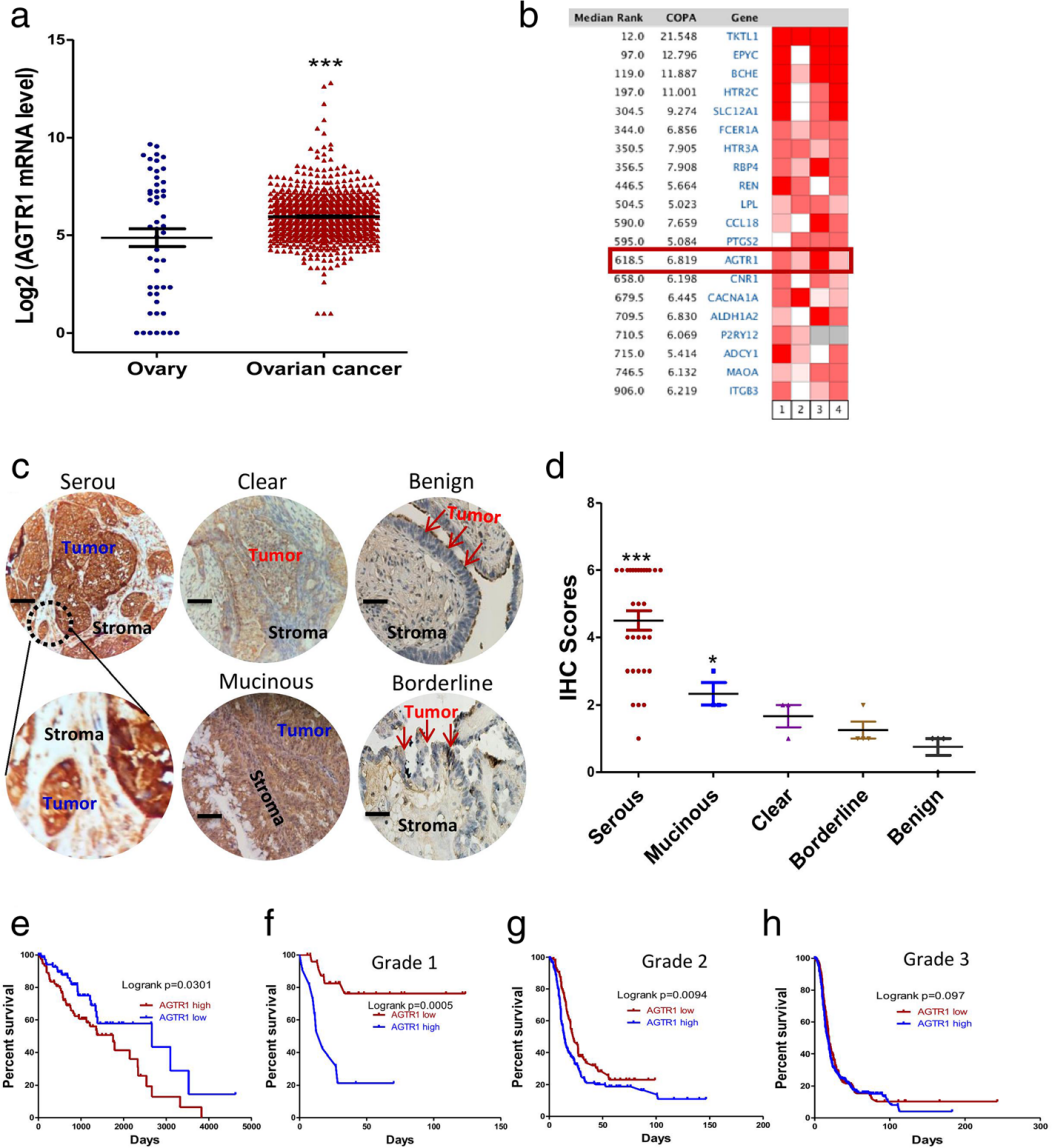
AGTR1 is upregulated in ovarian cancer tissues and negatively associated with prognosis. **a** AGTR1 mRNA expression profile in tissues of ovarian cancer (*n* = 902) and healthy controls (*n* = 50). The data were extracted from the GENT database. The data are presented as means ± SEM, *** *p* < 0.001, Student’s t-test. **b** AGTR1 mRNA expression patterns for four independent ovarian cancer studies (GSE12172, GSE26712, GSE12471, and TCGA ovarian carcinoma project). **c** Representative IHC staining images of AGTR1 from different pathological subtypes of ovarian cancer (Serous *n* = 30, Mucinous n = 3, Clear n = 3, Borderline *n* = 4, Benign n = 4), Scale bar = 50 μm. **d** IHC scores for different pathological types were assessed according to AGTR1 cellular location and signaling intensity. **e** Kaplan–Meier survival analysis based on AGTR1 expression levels in ovarian cancer patients (log-rank test). Patients with top 25% expression level of AGTR1 are defined as the AGTR1 high group and the bottom 25% patients are defined as the AGTR1 low group. Median overall survival (OS) 41.63 vs 57 months, *n* = 294, *p* = 0.0301). **f-h** The Kaplan–Meier survival analysis (log-rank test) based on cancer grade of the patients. The higher expression level of AGTR1 had poor outcomes in the Grade 1 (OS 18.1 vs > 120 months, *n* = 37, *p* = 0.0005) and Grade 2 (OS 19.5 vs 25.3, *n* = 256, *p* = 0.0094) patients, but was not significant for Grade 3 patients (*n* = 1015, *p* = 0.097)

To validate the AGTR1 expression profile in ovarian cancer patients and to assess its role in ovarian cancer progression, AGTR1 expression was assessed in 902 tumor tissues of ovarian cancer patients and 50 normal ovary tissues from the GENT database. AGTR1 expression in ovarian cancer tissues was higher than in normal ovary tissue (Fig. 1a). An outlier analysis tool was used to assess the data from four independent studies with a total of 1120 ovarian cancer patients. The results were consistent with the GENT database in that AGTR1 was generally upregulated in ovarian cancer patients of four different studies (Fig. 1b). To confirm AGTR1 expression in ovarian cancer tissues, IHC was performed on different pathological subtype of ovarian carcinoma. Representative samples of IHC staining for AGTR1 from different tissues are shown in Fig. 1c. IHC is scored from 0 to 6+, as a measure of AGTR1 level in ovarian cancer tissue samples. A score of 0 is defined as “negative for AGTR1”. A score of 1+ is defined as “equivocal for AGTR1”, while scores from 2+ to 6+ are “positive for AGTR1”. According to this scoring system, the frequency of positive staining for AGTR1 was higher in the serous and mucinous subtypes than in the other subtypes. Serous ovarian carcinoma showed very strong expression of AGTR1 (29/30 samples were positive). The expression of AGTR1, according to the average IHC score, in serous (*p* < 0.001) and mucinous (*p* < 0.05) subtypes was statistically higher than other subtypes (Fig. 1d). Moreover, we confirmed a correlation of AGTR1 expression with patient outcomes by using the TCGA RNA-seq data set that contains 294 ovarian cancer samples. The Kaplan–Meier survival analysis suggested that high AGTR1 expression was negatively correlated with survival time of ovarian cancer patients (Fig. 1e). Analysis by tumor grade showed that a significantly poorer prognosis was associated with high expression of AGTR1 when compared to lower tumor grade patients. High expression of AGTR1 predicted a shorter survival time for Grade 1 and Grade 2 tumor patients. While for tumor Grade 3, high expression AGTR1 did not increase death risk (Fig.1 f-h).

### ANGII significantly increases cell proliferation in a 3D spheroid model, but not in cells cultured in monolayer

Uncontrolled cell proliferation is the most important property of cancer cells. The effect of ANGII on the proliferation of ovarian cancer cells was assessed by the MTT assay. Multiple cell lines (Ovca429, HM, and A2780) were treated with ANGII. Similar to previous reports with the A2780 and HeyA8 cell lines [26], ANGII only slightly increased cell proliferation of monolayer cultures (Additional file 2: Figure S1a-c).

**Fig. 2:**
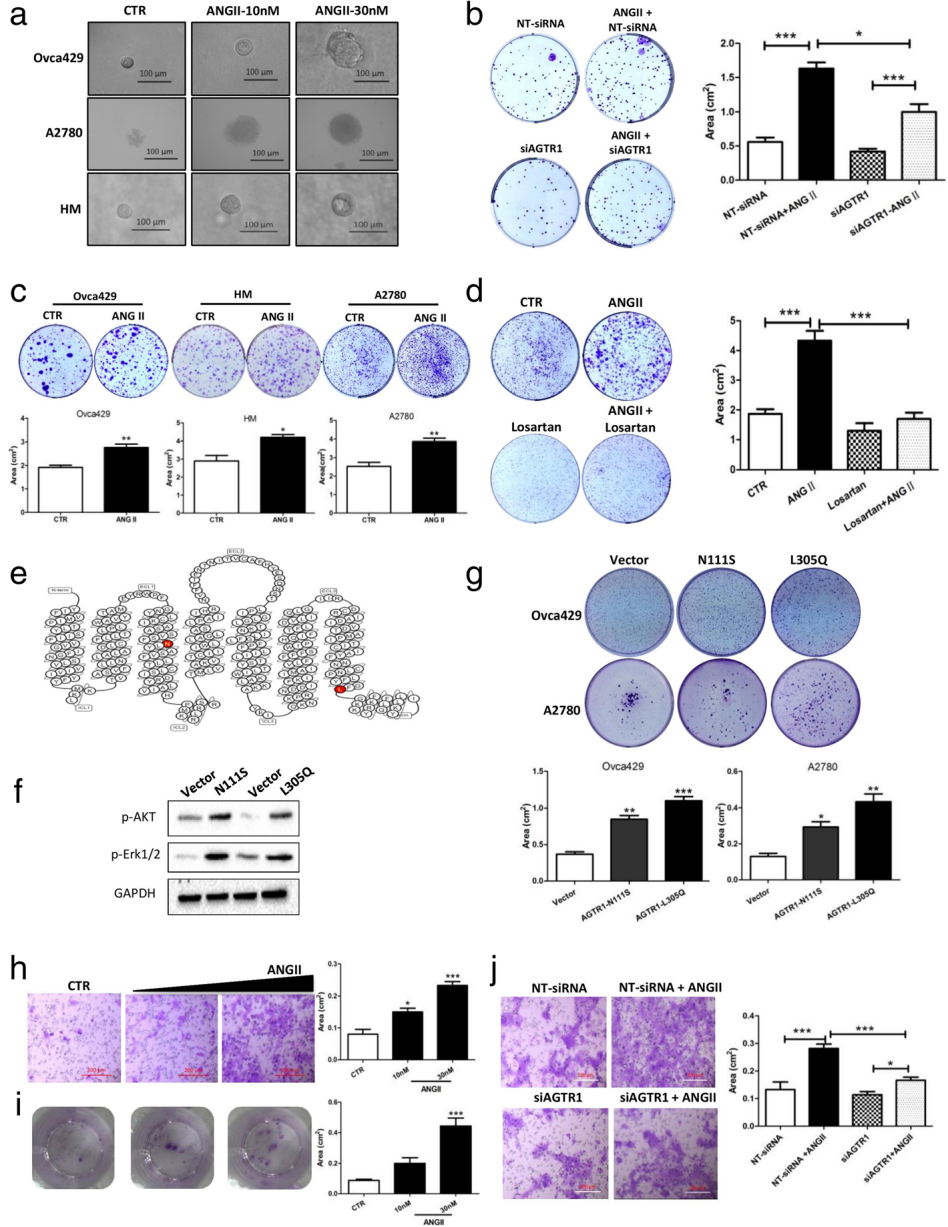
ANGII promotes ovarian cancer MCS formation and cell migration. **a** Bright field images of ovarian cancer spheroids by Ovca429, A2780, and HM cells. The images were captured after the cancer spheroids were cultured in Matrigel for 10 days. Scale bar, 100 μm. **b** The endogenous level of AGTR1 was knock-downed by siRNA (siAGTR1, 20 nM; Smartpool siRNA, E-005428-00, GE Dharmacon). The non-targeting siRNA (NT-siRNA, 20 nM; D-001910, GE Dharmacon) was used as a control. After transfection, cell proliferations of ovarian cancer spheroids was recorded and the growth area quantified. **c** ANGII (10 nM, 30 nM) enhanced MCS formation. Colonies formed by MCS were assessed by crystal violet staining. **d** The AGTR1 antagonist, losartan (10 μM), was used to inhibit the pathway and spheroid formation evaluated. **e** Construction of constitutively activated mutations of AGTR1. The positions of the constitutively activated mutations (AGTR1-CAM; N111S and L305Q) of AGTR1 are highlighted in red. **f** CAM directly activates the ERK/AKT pathway, even without ANGII. The phosphorylation of AKT and ERK1/2 in the AGTR1-CAM expressing Ovca429 cell line was confirmed by western blotting. The empty pcDNA3.1 stably transfected cell line was used as a control. **g** Spheroid formation by AGTR1-CAM expressing Ovca429 and A2780 cells was tested. There were significant increases in spheroid formation in AGTR1-CAM expressing cells. Upper: pictures of stained spheroids; lower: quantification of growth areas. **h** Transwell assays were performed to test the migration of Ovca429 cells after ANGII treatment (10 nM or 30 nM). Left: The cells on the bottom side of the transwell membranes were observed with a microscope. Scale bar, 200 μm. Right: The stained cell area was quantified by ImageJ software (at least five fields for each group). (**i**) Left: The migrated cells on the plates after 3 days of growth were stained with crystal violet. Right: The cell area was recorded and analyzed with ImageJ software. Scale bar, 100 μm. **j** siAGTR1 (20 nM) was transfected into Ovca429 cells to silence endogenous AGTR1 expression. Cell migration was assessed by transwell assay after silencing of AGTR1. The same amount of non-targeting siRNA (NT-siRNA) was used as a control. Left: images from the transwell membrane. Scale bar, 200 μm. Right: The cell area covered by the stained cells. The data are presented as means ± SEM. Significant differences compared to control are indicated with asterisks (* *p* < 0.05, *** *p* < 0.001)

MCS are commonly found in ascites and their stemness features enable cellular resistance to chemotherapy, increasing treatment complexity [27]. Growing tumor spheroids in vitro can partially mimic the in vivo tumor growth. These mimics are useful for cancer research and drug testing. To mimic the ovarian cancer cell growth within the peritoneum, a 3D cell model was employed. After ANGII treatment, maximum spheroid diameters were significantly increased for Ovca429, A2780, and HM cells when compared to control, with 3.37-, 2.72-, and 2.01-fold increases, respectively (Fig. 2a and Additional file 2: Figure S2a). To compare cell proliferation rates within spheroids, cells from spheroids were collected, reseeded, and cell growth area measured by crystal violet staining. The results demonstrated a dose-dependent increase in cell proliferation inside the spheroids after ANGII treatment, with a growth area increase (Additional file 2: Figure S2b). To confirm that AGTR1 mediates these ANGII effects, siRNA was used to silence AGTR1 expression. AGTR1 expression by different ovarian cell lines was measured by RT-qPCR with results indicating that Ovca429 had the greatest expression (Additional file 2: Fig. S3a). Therefore, Ovca429 cells were used for silencing experiments. AGTR1 siRNA significantly suppressed AGTR1 expression and reduced the AGTR1 protein level in Ovca429 (Additional file 2: Figure S3b&c). Results demonstrate that silencing of AGTR1 expression significantly reduces the effect of ANGII on spheroid growth in the 3D culture model (Fig. 2b).

### ANGII enhances multicellular spheroid formation

MCS formation is a key step in transcoelomic spread of ovarian cancer cells within the peritoneal cavity [28]. Further, MCS formation also enhances the chemotherapy resistance of cancer cells [29]. More than just promoting the growth of cells, we propose that ANGII increases MCS formation by cancer cells, which promotes metastasis of ovarian cancer. Hence, a role for ANGII and AGTR1 in MCS formation was assessed with three ovarian cancer cell lines; Ovca429, A2780, and HM. MCS formation was significantly enhanced by ANGII for each of the cell lines (Fig. 2c). The effect of ANGII was significantly reduced by the AGTR1 antagonist, losartan (Fig. 2d). Two AGTR1 constitutively-active mutations (CAM) stable cell lines (N111S and L305Q) were assessed for cell signaling and spheroid formation (Fig. 2e). For both of the CAM cell lines, an increase in the phosphorylation of AKT (Ser473) and ERK1/2 (Thr202/Tyr204) was demonstrated, indicating activation of the PI3K/AKT and the MEK/ERK1/2 signaling pathways in the AGTR1-CAMs (Fig. 2f and Additional file 2: Figure S2c). For spheroid formation, Ovca429 cells with AGTR1-CAMs expression formed MCS more easily than the empty vector control. Spheroid growth areas of AGTR1-CAM cell lines were significantly greater than controls (Fig. 2g). In summary, these data demonstrate ANGII to stimulate MCS formation through AGTR1, rather than through other receptors, such as AGTR2.

### ANGII accelerates the migration of ovarian cancer cells

To examine whether ANGII enhances ovarian cancer cell migration, we performed a transwell migration assay with Ovca429 cells. The data show the number of migrated cells (cells across the membrane and cells that migrated into the well) were significantly increased after ANGII treatment (Fig. 2h-i). Suppression of AGTR1 levels by siRNA essentially abolished the effect of ANGII treatment (Fig. 2j). After cell migration to the bottom of the well, cell morphology was different from parental cells. The migrated cells were more mesothelial than epithelial (Additional file 2: Figure S2d). Furthermore, the expression of AGTR1 and angiotensinogen (AGT, the precursor gene of ANGII) in the migrated cells was higher than in parental cells (Additional file 2: Figure S2e). This increase in AGTR1, in the migrated cells, is in agreement with the database, indicating a correlation between AGTR1 and cancer prognosis. That is, AGTR1 is expressed at a higher level in highly metastatic “mesenchymal subtype” ovarian cancer tissues (Additional file 2: Figure S4a).

### ANGII triggers the ERK1/2 and AKT pathways as well as transactivates the EGFR signaling pathway of ovarian cancer cells

**Fig. 3:**
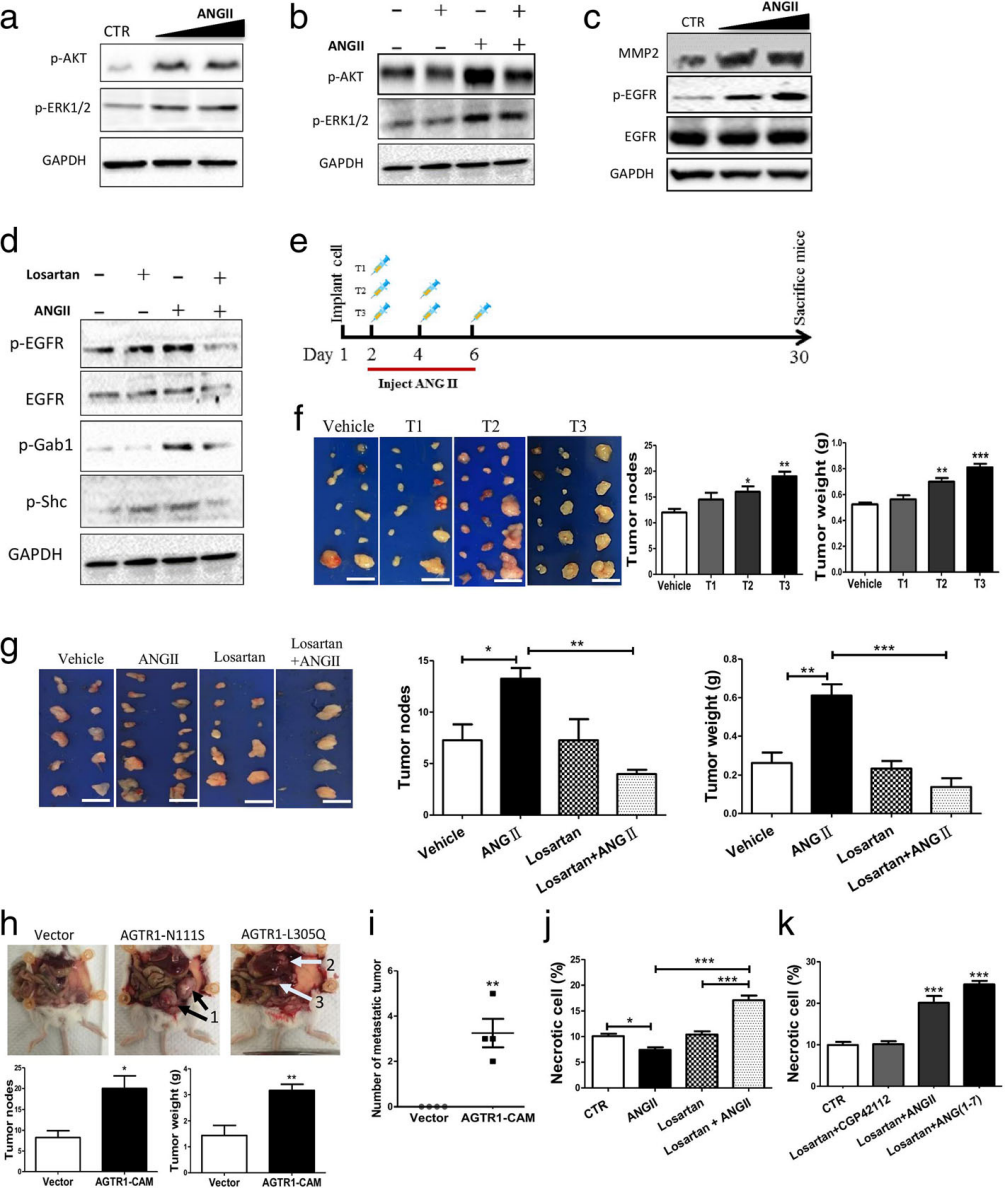
ANGII triggered classical AGTR1 signaling and the transactivation of EGFR in ovarian cancer cells that resulted in cancer dissemination inside the abdominal cavity. **a** Activation of the classical AGTR1 pathway. Western blotting of p-AKT and p-ERK1/2 with or without ANGII treatment. GAPDH is the loading control. **b** Losartan suppressed the activation of the AKT/ERK pathway by ANGII. Immunoblots of p-AKT and p-ERK1/2 with ANGII and/or losartan treatment. **c** ANGII transactivates EGFR in a ligand-dependent manner. Western blotting of MMP2, EGFR, and p-EGFR, with GAPDH as a control. **d** Losartan suppressed the ANGII-mediated transactivation. Western blotting of p-EGFR, EGFR, p-Gab1, p-Shc, and GAPDH. **e** Schematic representation of ANGII administration to the animals. **f** Left: tumors inside the peritoneal cavity were dissected and representative images presented. Right: the number and weight of tumor nodes inside the peritoneal cavity after ANGII (1 μg/kg) treatment (each group *n* = 6). **g** Administration of losartan (40 μg/kg) reduced the effect of ANGII (100 nM) on ovarian cancer cell development inside the peritoneal cavity of mice. Upper: tumors removed from the peritoneal cavity with representative images for each group (each group n = 6). Lower: the number of tumor nodes and tumor weights. **h** AGTR1-CAM strongly enhanced ovarian cancer cell metastasis inside the peritoneal cavity. AGTR1-CAM cell lines were i.p. injected into NOD-SCID mice and were evaluated after 30 days (*n* = 4). Upper: tumor metastasis into a new organ inside the peritoneal cavity (“1” indicates metastatic tumor inside an ovary; “2” indicates metastatic tumor in the liver; “3” indicates metastatic tumor in the spleen). Lower: the number of tumor nodes and tumor weight in the peritoneal cavity of mice. **i** The number of metastatic tumors in the mice. **j** Cell death of MCS was assessed by Annexin V-FITC and PI assay by flow cytometry after treatment with ANGII (100 nM) and/or losartan (10 μM). Necrotic cells in each group were quantified. **k** Cell necrosis inside MCS was detected by flow cytometry with different combinations of treatment: ANGII (100 nM), losartan (10 μM), CGP42112 (50 nM) and/or ANG(1–7) (100 nM). Necrotic cells in each group were quantified accordingly. All of the data in this figure are presented as means ± SEM from at least three experiments. Significant differences are indicated (* *p* < 0.05, ** *p* < 0.01, *** *p* < 0.001 against control)

To determine the signaling mechanism for ANGII in ovarian cancer cells, the related signaling pathways, MAPK/ERK1/2 and PI3K/AKT, were assessed. These pathways are well-known to be activated by the ANGII/AGTR1 axis in the cardiovascular system and as well to stimulate cell proliferation, survival, and metastasis in other cancer models. The data herein demonstrates ANGII to phosphorylate ERK1/2 (Thr202/Tyr204) and AKT (Ser473) (Fig. 3a and Additional file 2: Figure S5a). Blocking AGTR1 with losartan reduced ANGII-mediated phosphorylation of ERK1/2 and AKT (Fig. 3b and Additional file 2: Figure S5b). These data suggest ANGII activation of the classical AGTR1-related pathways in ovarian cancer cells.

Transactivation of EGFR by G protein–coupled receptors (GPCRs) is widely believed to be an important physiological process for cancer development, although the importance of GPCRs in ovarian cancer is not well understood. Herein, EGFR transactivation by ANGII was assessed. ANGII increased MMP2 expression as well as phosphorylation of EGFR at Tyr1068 (Fig. 3c and Additional file 2: Figure S5c). MMPs function to release EGFR ligands. Hence, ANGII transactivation of the EGFR signaling pathway in the ovarian cancer cells is a ligand-dependent mechanism. In addition, suppression of AGTR1 by losartan significantly suppressed this transactivation as shown by reduced EGFR phosphorylation (Fig. 3d and Additional file 2: Figure S5d). ANGII also stimulated the phosphorylation of downstream transducers of the EGFR signaling pathway, Gab1 and Shc. This stimulation was reversed by losartan treatment (Fig. 3d and Additional file 2: Figure S5d). These data confirmed the involvement of ANGII and its receptor in ligand-dependent transactivation of the EGFR pathway.

Using the TCGA database, clinical data from 606 ovarian cancer tissues were analyzed. Protein profiles from AGTR1-high and AGTR1-low patient tissues were assessed. As expected, the levels of p-ERK1/2, p-AKT (Ser473), and EGFR were higher in the AGTR1-high patient tissues than in the AGTR1-low cases (Additional file 2: Figure S6a). The top-tier pathway identified by GO analysis was the EGFR signaling pathway. Others identified pathways were the HIF-1 signaling pathway and the negative regulation of cell apoptosis, both pathways well-known to be involved in cancer progression (Additional file 2: Figure S6b). In summary, these in vitro data and the bioinformatics analysis of patient tissues are consistent, suggesting that AGTR1 activates the ERK and AKT pathways as well as the transactivation of the EGFR pathway.

### Activation of the AGTR1 pathway promotes ovarian cancer tumor formation and metastasis within the peritoneal cavity

Xenograft experiments with Ovca429 cells in NOD-SCID mice assessed the function of ANGII in vivo with regard to peritoneal metastasis. By increasing the number of ANGII administrations (from 1 to 3 times), significant increases in tumor node number and tumor weight were found (Fig. 3e-f). Losartan prevented peritoneal colonization and spreading of the tumor (Fig. 3g). Since i.p. injected ANGII may reach the peripheral organs, the observed effects of ANGII may be due to the activation of the AGTR1 pathway in peripheral organs, rather than by direct activation of ovarian cancer cells. To exclude this possibility, AGTR1-CAM cell lines were assessed and as shown in Fig. 3h, mice inoculated with AGTR1-CAM ovarian cancer cells had more and larger tumor nodes when compared to control. More important, the AGTR1-CAM cells formed metastatic tumor in the stomach, liver, and spleen (Fig. 3i).

Interestingly, the sole use of losartan did not significantly reduce tumor node number or weight, but the combined treatment of ANGII with losartan did when compared to control (Fig. 3g). This result suggests the potential involvement of an alternative pathway(s). Based on this hypothesis, we analyzed cell death within the ovarian cancer spheroids. MCS were treated with ANGII and/or losartan before Annexin V analysis. Treatment with ANGII or losartan alone did not alter the level of apoptosis, but co-treatment with ANGII and losartan significantly increased the number of necrotic cells (Fig. 3j and Additional file 2: Figure S7a). Therefore, we proposed that ANGII converted into another form, such as ANG(1–7), and/or interacted with other receptors (AGTR2 or MAS1) in the renin-angiotensin system (RAS) to promote cancer necrosis when the AGTR1 pathway was blocked. ANGII can interact with AGTR2. Further, ANGII can activate MAS1 after conversion to ANG(1–7) by Angiotensin-converting enzyme 2 (ACE2). Hence, co-treatment with losartan was conducted with the AGTR2 agonist, CGP42112, and the MAS1 agonist, ANG(1–7). Losartan and ANG(1–7), but not CGP42122 triggered a similar necrotic response as with ANGII/losartan co-treatment (Fig. 3k and Additional file 2: Figure S7b), indicating a counter-acting mechanism between MAS1 and AGTR1 in ovarian cancer cells.

### In vitro and in vivo release of ANGII from ovarian cancer cells

**Fig. 4:**
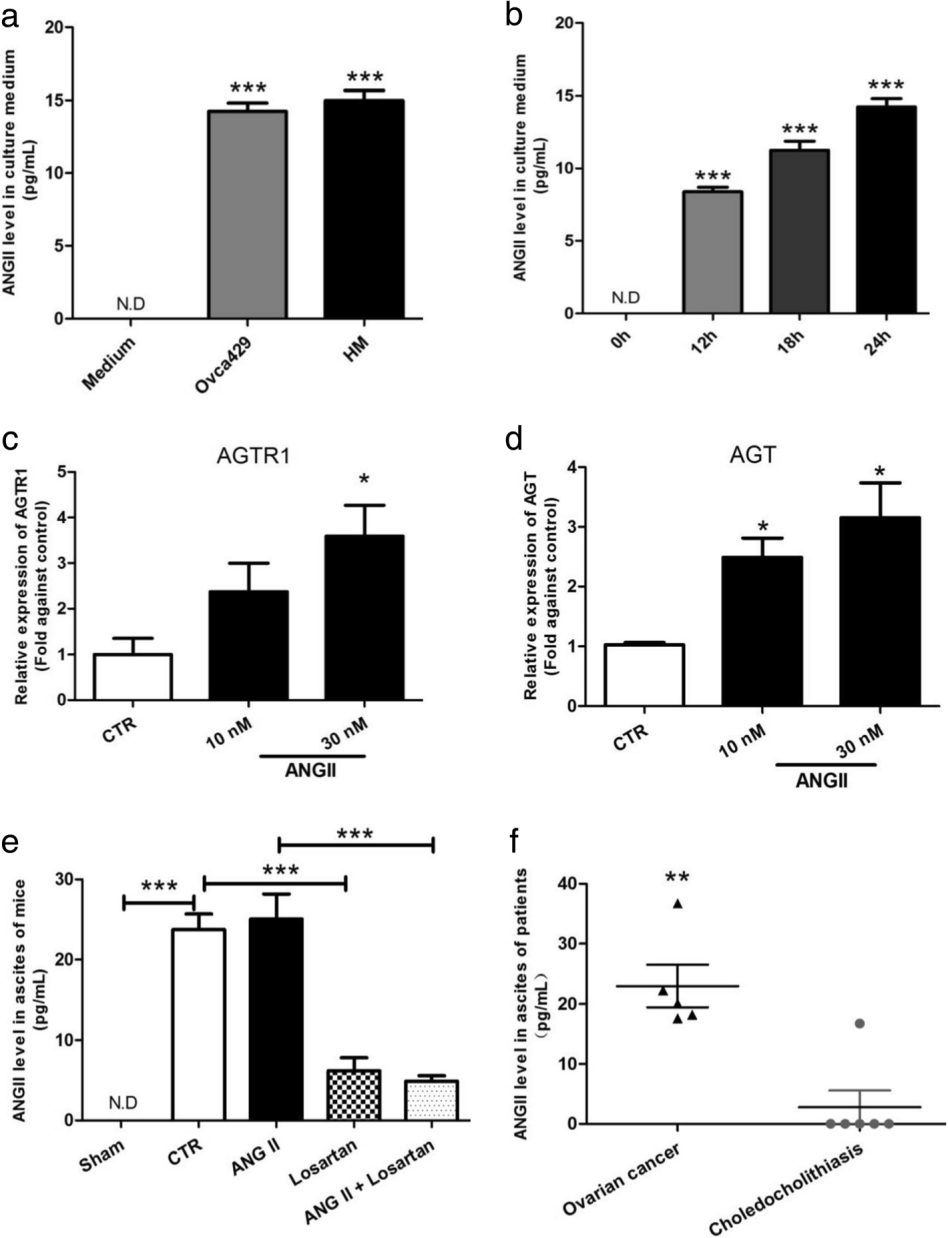
ANGII and AGTR1 formed a positive feedback loop that enhanced ovarian cancer progression. **a** Secretion of ANGII by ovarian cancer cells (Ovca429 and HM) into the culture medium. The ANGII level was measured by enzyme immunoassay. **b** Accumulation of ANGII in the culture medium of Ovca429 cell was measured. **c** & **d** AGTR1 and AGT mRNA expression after ANGII treatment, which ranged from 0 to 30 nM and was measured by RT-qPCR after ANGII treatment. (**e)** ANGII levels in ascites of xenografted mice (each group n = 4). Sham: mice injected with Hanks’ buffer without cancer cells; CTR: mice inoculated with cancer cells without drug administration; ANGII: mice inoculated with cancer cells and treated with ANGII (100 nM) at days 1, 3, and 5; ascites was collected at day 30. Losartan: mice inoculated with cancer cells and treated with losartan (10 μM), ANGII+losartan: mice inoculated with cancer cells and treated with ANGII (100 nM) and losartan (10 μM) (**f**). ANGII levels in ascites of ovarian cancer patients (*n* = 5) and choledocholithiasis patients (n = 6). The data are presented as means ± SEM from at least three experiments; * *p* < 0.05, ** *p* < 0.01, *** *p* < 0.001 against choledocholithiasis patients

Since there is a significant increase in AGT after ANGII treatment, we hypothesized that ovarian cancer cells directly secrete ANGII enhancing MCS formation and growth, in turn, to increase cancer metastasis in a positive feedback manner. To verify this hypothesis, we measured ANGII levels in the culture medium of Ovca429 and HM cells. High ANGII levels (Ovca429, 14.23 ± 0.8 pg/mL; HM 14.98 ± 1.0 pg/mL) were found when compared to complete medium with 2% FBS (Fig. 4a). In addition, there was a time-dependent increase in ANGII levels as incubation time increased (Fig. 4b). These data demonstrate ANGII secretion by ovarian cancer cells. In addition, the expression levels of AGTR1 and AGT after ANGII treatment were measured. Consistent with previous data, ANGII increased the expression of AGTR1 and AGT in a dose-dependent manner (Fig. 4c-d). In addition to the in vitro model, a high level of ANGII (23.76 ± 3.3 pg/mL) was also detected in ascites collected from ovarian cancer cell inoculated xenografted mice. ANGII was not detected in peritoneal fluid from control mice. Although ANGII administration did not further increase ANGII levels, losartan administration did reduce ANGII in ascites (Fig. 4e). Analysis of ascites from ovarian cancer patients and noncancerous (choledocholithiasis) patients demonstrated ANGII concentration to be higher in cancer patient’s ascites than in noncancerous patients (22.9 ± 7.9 pg/mL vs 2.79 ± 6.8 pg/mL) (Fig. 4f). In summary, data from in vitro cell lines, the xenograft models, and patient samples demonstrate ANGII secretion by ovarian cancer cells. Since ANGII promotes MCS growth and metastasis of ovarian cancer cells, it is likely that ANGII and AGTR1 form a positive feedback loop that enhances ovarian cancer malignancy.

### ANGII promotes lipogenesis and lipid desaturation to suppresses ER stress in MCS

**Fig. 5:**
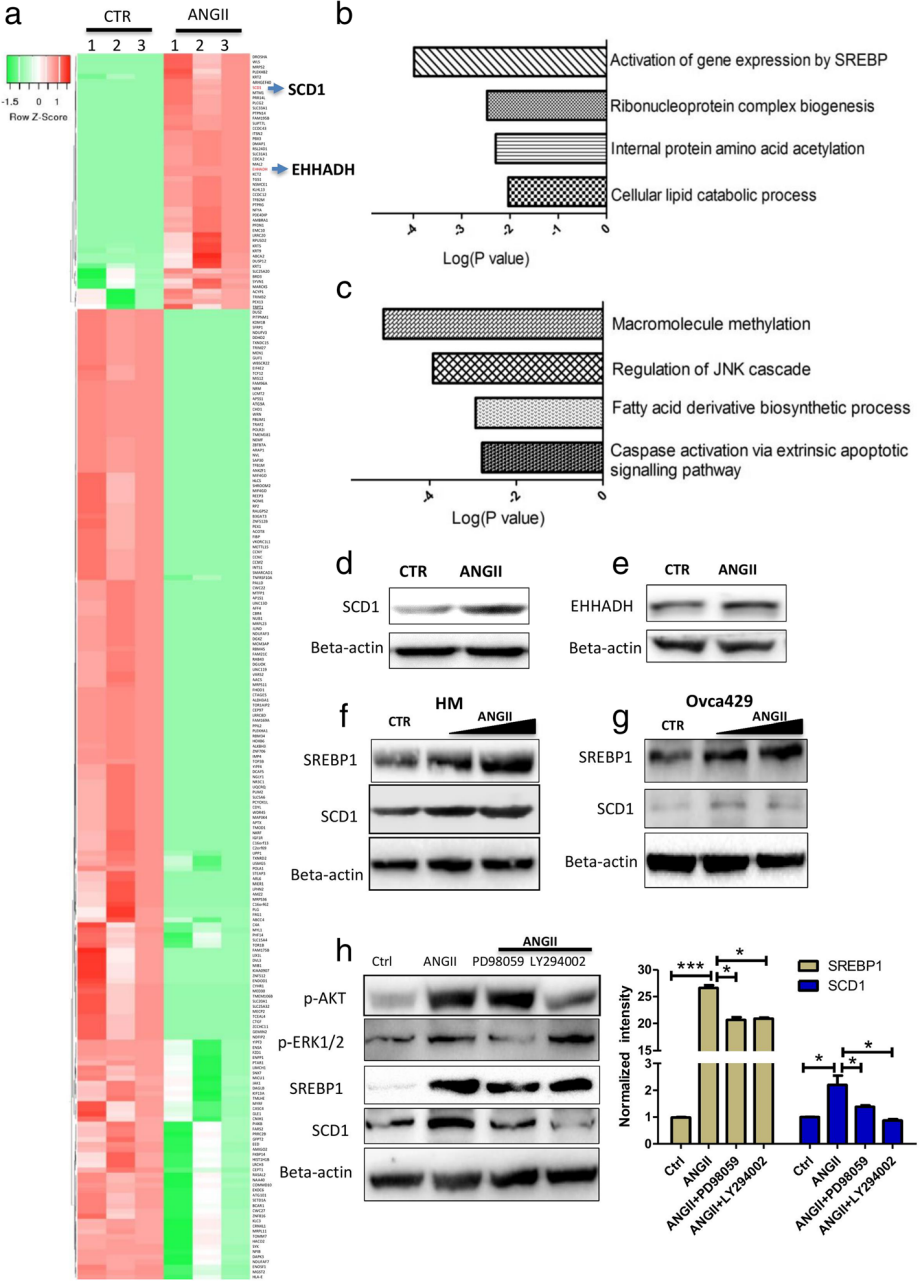
ANGII enhanced lipid metabolism and promoted MCS formation. **a** Based on proteomic analysis, differentially expressed proteins after ANGII treatment are presented by heatmap. **b** GO enrichment analysis was employed to analyze the biological function of the upregulated proteins. **c** GO enrichment analysis was employed to analyze the biological function of the downregulated proteins. **d** and **e** Changes in SCD1 and EHHADH protein levels after treatment with ANGII (30 nM) confirmed by western blotting. **f** The expression of SCD1 and SREBP1in HM cells after ANGII treatment measured by western blotting. **g** The expression of SCD1 and SREBP1 after ANGII treatment in Ovca429 cell were measured by western blotting. **h** Western blotting confirmed ANGII induced-SCD1 expression was suppressed by blocking of MEK/ERK1/2 and PI3K/AKT pathways. The data are presented as means ± SEM from at least 3 experiments and significant difference are indicated (* *p* < 0.05, *** *p* < 0.001 against control)

To explore the molecular mechanistic basis for ANGII promotion of ovarian cancer metastasis, a shotgun proteomic approach was used to identify protein changes in ovarian cancer cells (Ovca429) after ANGII treatment. The proteomic analysis identified 6155 proteins with 50 upregulated proteins and 190 down-regulated proteins (FC > 2 and *p* < 0.05) (Fig. 5a and Supplementary Table S3). GO analysis, performed on the upregulated proteins, identified four functional clusters. The top-tier function was “Activation of gene expression by SREBP” (Fig. 5b). Multiple SREBP pathway related genes were upregulated, including NFYA, SCD1, TGS1, PLCG2, AMBRA1, EHHADH, and MTM1 (Supplementary Table S3). Similarly, GO analysis, performed on the down-regulated proteins demonstrated suppression of the JNK cascade and the caspase related extrinsic apoptotic cell signaling pathway (Fig. 5c). Proteomic data was verified by western blotting. As shown in Fig. 5d and e, the expression levels of Stearoyl-CoA Desaturase-1 (SCD1), noyl-CoA Hydratase, and 3-Hydroxyacyl CoA Dehydrogenase (EHHADH) were upregulated by ANGII treatment, consistent with the proteomic data (Additional file 2: Figure S8a and b). Sterol regulatory element-binding protein (SREBP) regulated genes are well known for their function in lipid homeostasis. Western blotting were carried out to verify the proteomic data. As shown in Fig. 5f and g, ANGII treatment significantly increased the SREBP1 and SCD1 expression in HM and Ovca429 cells. We have demonstrated that ANGII trigger MEK/ERK1/2 and PI3K AKT pathway. To elucidate the role of these pathway in activating the SREBP1, blockers of ERK an AKT pathways (PD98059 and LY294002) were used. Inhibition of ERK1/2 and AKT pathways could significantly impair the ANGII-mediated gene expression of SREBP1 and SCD1 (Fig. 5h and Additional file 2: Fig. S8e).

**Fig. 6:**
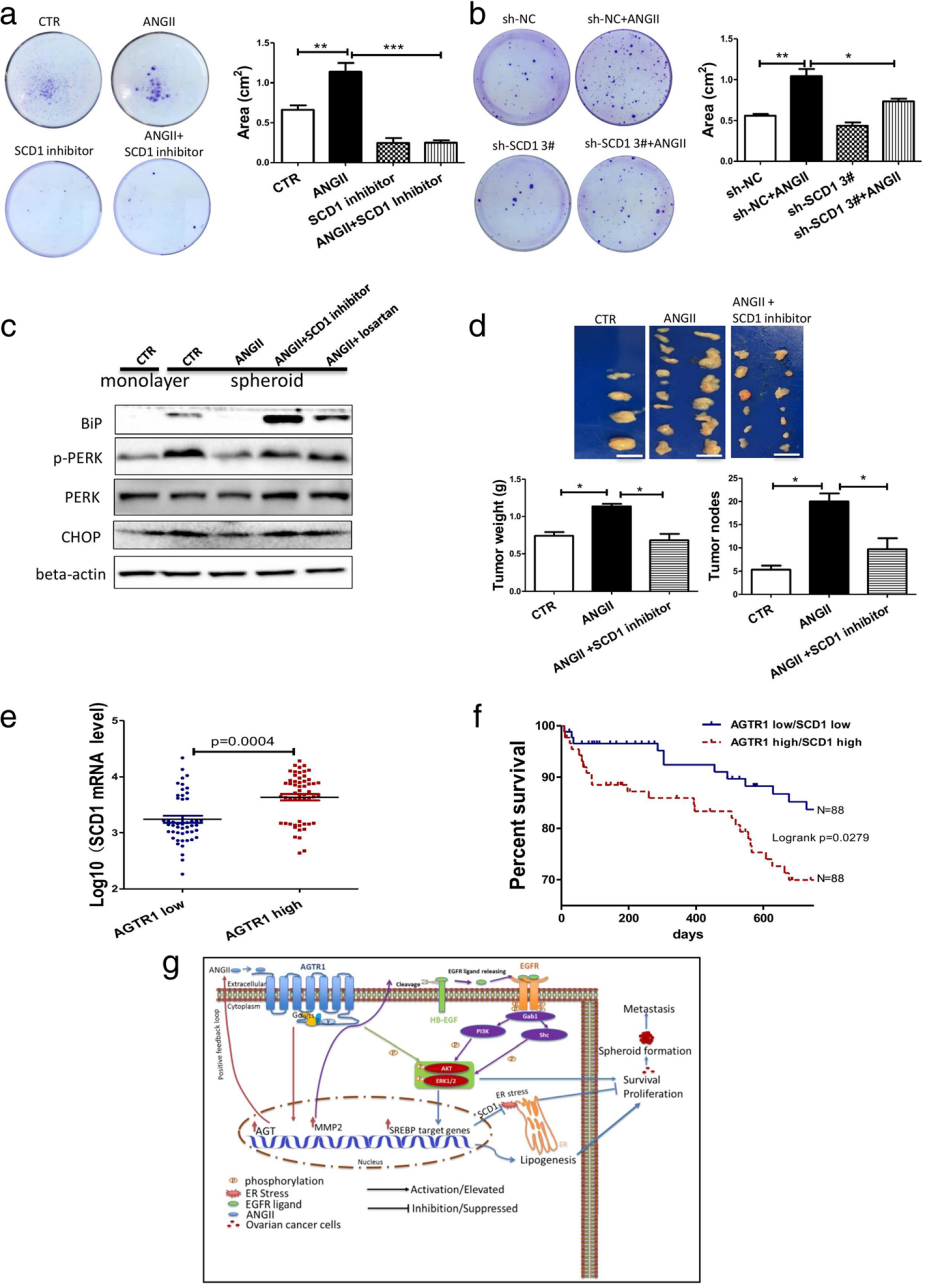
ANGII induced SCD1 expression resulted in alleviation of ER stress in MCS. **a** SCD1 inhibitor was employed to block the ANGII effect on spheroid formation. The data are presented as means ± SEM from at least three experiments with significant differences against control indicated (HM cells, ** p < 0.01,*** p < 0.001). **b** shRNA vector was used to silence the SCD1 expression and verify the involvement of SCD1 in the ANGII induced spheroid formation. The data are presented as means ± SEM from at least three experiments with significant differences against control indicated (HM cells, * *p* < 0.05, ** *p* < 0.01). **c** Necrotic marker, BiP, CHOP, and p-PERK expression in MCS was increased during MCS formation, but suppressed by ANGII treatment and reversed by the SCD1 inhibitor. Protein levels of BiP, CHOP, and p-PERK after various treatments, quantified by western blot. **d** SCD1 inhibitor (20 μg/kg) was used to block the ANGII effect on tumor formation and cancer metastasis in xenografted mice. Tumor nodes and tumor weights were and measured. The data are presented as means ± SEM (*n* = 3 for each group, * *p* < 0.05 against the corresponding control group). **e** SCD1 mRNA expression levels in AGTR1 high expression patients and AGTR1 low expression patients. AGTR1 expression levels belonging to the lower tertile were defined as the “AGTR1 low” group and the upper tertile defined as the “AGTR1 high” group. **f** Survival expectation of patients with different expression patterns of AGTR1 and SCD1 were assessed by Kaplan-Meier analysis. Logrank analysis was performed to compare the difference between AGTR1-Low/SCD1-Low group and the AGTR1-High/SCD1-High group. **g** Schematic diagram summarizing the molecular mechanism for ANGII/AGTR1 enhanced the ovarian cancer MCS formation, MCS growth, and metastasis. Arrows in red: the positive feedback loop of ANGII/AGTR1 in ovarian cancer cells. Arrows in green: the classic Gq dependent signaling pathways. Arrows in purple: the transactivation of EGFR by AGTR1. Arrows in blue: the SREBP pathways increase lipogenesis and suppress ER stress, enhancing MCS formation and metastasis

Among the genes in this pathway, we were particularly interested in SCD1. SCD1 not only participates in fatty acid desaturation but may also be involved in cancer stem cell (CSC) maintenance [30, 31]. Hence, SCD1 may mediate the ANGII effect on the spheroid formation and may prevent necrosis during spheroid formation. To test this hypothesis, we used a SCD1 inhibitor (ab142089, Abcam) to block the related pathway. As expected, the SCD1 inhibitor treatment almost completely abolished the ANGII-mediated formation and growth (Fig. 6a). Furthermore, the SCD1 inhibitor also significantly induced the necrosis of MCS even after ANGII treatment of HM and Ovca429 cells (Additional file 2: Figure S9a and b). To confirm the role of SCD1 in the ANGII-mediated MCS formation, shRNAs were used to knockdown SCD1. Four shRNA plasmids were used; all shRNAs significantly silenced SCD1 expression (Additional file 2: Figure S9c). In the spheroid formation assay, the knockdown of SCD1 dramatically reduced the effect of ANGII on ovarian cancer MCS formation (Fig. 6b and Additional file 2: Figure S9d). It is well known that the growth and formation of MCS are associated with an increase in ER stress [32, 33]. Moreover, previous reports have suggested a relationship between SCD1 and cell necrosis, in that SCD1 promotes the formation of necrotic cores in the proximal aorta [34] and SCD1 inhibitors induce necrosis of hepatocellular carcinomas [35]. Hence, we hypothesized that reduction in MCS necrosis by ANGII is related to ER stress and that SCD1 plays a key role in this pathway. PERK is a receptor in the ER membrane which monitors ER stress. Binding immunoglobulin protein (BIP) and CHOP are ER stress-response protein [36]. Increasing phosphorylation level of PERK and stress-response protein reflect cellular ER stress status [32]. SCD1 is a lipid desaturase, that introduces double bonds into fatty acid in order to maintain membrane fluidity, reducing ER stress during unfavorable conditions [37]. As showed in Fig. 6c, MCS formation significantly increased BiP, CHOP levels as well as p-PERK. In contrast, BiP, CHOP, and p-PERK levels were dramatically reduced in spheroid cells after ANGII treatment. This effect of ANGII was reversed with the SCD1 inhibitor losartan. These data strongly suggest that ANGII alleviates ER stress-induced necrosis via modulation of SCD1 levels. The role of SCD1 in vivo was assessed by injection of the SCD1 inhibitor into an ovarian cancer, peritoneal-xenograft mouse model. The co-injection of the SCD1 inhibitor partially reduced the effect of ANGII on ovarian cancer cell peritoneal-cavity dissemination (Fig. 6d).

In addition, SCD1 expression levels were compared between “AGTR1-high” and “AGTR1-low” patients. There was a strong positive correlation between AGTR1 and SCD1 expression levels in ovarian cancer patients (Fig. 6e). Regarding patient outcomes, the patients with high expression levels of AGTR1 and SCD1 had significantly lower survival rates when compared to the patients with low levels of both AGTR1 and SCD1 (Fig. 6f). In conclusion, ANGII upregulates SCD1, which increases the lipid desaturation and relieves ER stress during MCS formation and growth, promoting ovarian cancer progression and metastasis.

## Discussion

ANGII has been reported to promote cell proliferation in monolayer cell cultures of cell lines A2780 and HEYA8. However, micromolar concentrations of ANGII (10 to 100 μM) were required [26]. Our data are consistent with that report in that ANGII at nanomolar concentrations (10 to 90 nM) only slightly increased proliferation of Ovca429, A2780, and HM cells. Therefore, we hypothesized that ANGII may have other functions that enhance ovarian tumor development other than direct stimulation of cancer cell proliferation. As ovarian cancer preferentially spreads and metastasizes through the peritoneal cavity, MCS formation is essential for ovarian cancer peritoneal metastasis [29, 38, 39]. Hence, we hypothesized that AGTR1 activation would promote tumorigenesis and peritoneal metastasis by enhancing MCS formation and MCS growth. ANGII was found, even at nanomolar concentrations, to significantly enhance MCS growth and spheroid formation (Fig. 2a &c).

In addition, ANGII was shown to stimulate ovarian cancer cell migration, with the migrated cells expressing higher AGTR1 than parental cells (Fig. 2h). Moreover, migrated cell morphology was more mesenchymal-like compared to parental cells (Additional file 2: Figure S2d). Therefore, ANGII may induce cancer stem cell (CSC) like properties and be more metastatic. Bioinformatics analysis confirmed this hypothesis. AGTR1 expression is positively correlated with epithelial-mesenchymal transition (EMT) scores (Additional file 2: Figure S4b). GSEA analysis supported the expression of an EMT gene set including; E-cadherin, N-cadherin, and TGF-beta. Each was upregulated in patients with high AGTR1 expression (Additional file 2: Figure S4c). Epithelial ovarian cancer (EOC) cells undergo EMT transition, which liberates cancer cells from the primary tumor site and allows migration to distant organs [40]. Furthermore, AGTR1 is highly expressed by the metastatic subtype (Additional file 2: Figure S4a) with high expression of AGTR1 associated with poor patient outcomes (Fig. 1). Taken together, these results suggest that higher expression of AGTR1 is likely to be greater in metastatic ovarian cancer cells.

Large spheroids consist of an outer proliferating region, an intermediate region of quiescent cells, and a necrotic core due to a lack of oxygen and nutrients [41]. Herein, the growth of the MCS dramatically increased necrotic cell death, but ANGII reduced necrosis (Fig. 3j). Interestingly, co-treatment with losartan and ANGII induced greater cell death than losartan alone. This result is similar to the tumor xenograft results in which co-treatment with losartan and ANGII resulted in a further decrease in both tumor volume and the number of tumor nodes (Fig. 3g). These results suggest that not only are signaling pathways regulated by AGTR1 responsible for tumorigenesis but also that other signaling pathways activated by ANGII or related products are involved in the suppression. From the RAS pathway, possible receptors are AGTR2 and MAS1 that have opposing effects to that of AGTR1 in several cancers [42–45]. Cell death analysis suggested MAS1 rather than AGTR2 to be involved (Fig. 3k). When losartan was combined with ANGII or ANG(1–7), the percentage of necrotic cells was greatly elevated compared to control. Combined treatment with losartan and CGP42112 had no effect. Activation of AGTR2 has been reported to reduce the effect of ANGII on ovarian cancer cell survival [26]. In this study, no significant effect of the AGTR2 agonist, CGP42112A, on cell death was observed. This may be due to the extremely low expression of AGTR2 compared to AGTR1 and MAS1 (Additional file 2: Figure S3d). Since ACE2 can cleave ANGII forming ANG(1–7) and since ANG(1–7) is able to activate the MAS1 receptor [46], co-treatment with ANGII and losartan enhanced the necrosis of ovarian cancer cells. This likely results from activation of MAS1 and the inhibition of AGTR1, reducing survival and growth of the spheroids as well as cancer metastasis.

Proteomic analysis revealed the molecular pathways by which AGTR1 enhances MCS formation and MCS growth (Fig. 5b). GO analysis suggested activation of the SREBP pathway. The SREBP pathway is well known to upregulate lipogenesis by increasing expression of various lipogenic enzymes including; FASN, ACC, and SCD1 [47]. For example, the growth of glioblastoma in xenograft models is sharply reduced if the SREBP pathway is inhibited [48, 49]. The hostile microenvironment of the peritoneal cavity does not favor unregulated growth of cancer cells, therefore cancer spheroids have an increased demand for fatty acids [50]. The findings herein suggest that the effect of ANGII on MCS and metastasis is via an increase in lipid synthesis. Instead of endogenous production of fatty acids from citrate, cancer cells up-take fatty acids from exogenous sources, such as adipocytes.

The unregulated growth of cancer cells increases ER stress and induces cell death. One of the reasons is the overload of saturated fatty acid results in lipotoxicity [39, 51, 52]. Therefore, proliferating cancer cells and/or hypoxic cells need to balance their growth rate and unsaturated lipid levels to prevent ER stress and to maintain cell survival [47]. This can be accomplished by increasing the rate of unsaturated fatty acid synthesis and the use of both endogenous and exogenesis fatty acids. SCD1 is a desaturase localized within the ER, which introduces double bonds into fatty acids [37, 53]. The data herein suggest that SCD1 is significantly increased after ANGII treatment (Fig. 5c). As reflected by the reductions in BiP and pPERK, ANGII may suppress the ER stress level by upregulation of SCD1. Hence, ANGII treatment would reduce cellular necrosis via an increase in unsaturated fatty acids within ovarian cancer spheroids. This may explain why ANGII only has a slight effect on cell proliferation in monolayer culture conditions. ER stress increases during tumor formation (or in cancer spheroid formation) due to the limited accessibility of nutrients and oxygen. ANGII could reduce the ER stress significantly by increases the production of unsaturated lipids. In contrast, culture conditions during anchorage-dependent growth are optimal and ER stress levels are relative low. Therefore, AGTR1 activation has only a minor effect on proliferation of cells cultured in monolayer.

Activation of PI3K/AKT and/or MAPK/ERK1/2 triggers the SREBP pathway and induces the expression of lipogenesis genes [54–56]. The results herein have demonstrated ANGII to trigger PI3K/AKT and MAPK/ERK1/2 signaling pathways (Fig. 3). Interestingly, transactivation of EGFR by GPCR has been reported in various cancers [57–59]. We also found that ANGII treatment increased the phosphorylation of EGFR with activation of downstream Gab1 and Shc proteins in ovarian cancer cells. Blocking of PI3K/AKT and MEK/ERK pathway reverse ANGII effect on ovarian cancer cell spheroid formation. Therefore, ANGII stimulation of the SREBP pathway is by activation of both the classical Gq signaling pathway as well as by EGFR transactivation. In addition to the SREBP pathway, the proteomic data suggest the suppression of JNK cascade transduction and inactivation by the extrinsic apoptotic cell signaling pathway. Both of the pathways are believed to mediate ER stress [60, 61] and may influence ER stress during MCS formation.

Exogenous ANGII has been reported to induce mesenchymal stem cell production of ANGII [62]. Herein, ANGII upregulated expression of its precursor gene, AGT, stimulating the release of ANGII into the culture medium (Fig.4a-b). In vivo, the concentration of ANGII in the xenograft mice was notably higher than control animals. These data strongly support our hypothesis that the ANGII/AGTR1 axis forms a positive feedback loop, enhancing the effect of ANGII on cell migration and MCS formation. Interestingly, ANGII in ascites was also found in severe ovarian hyper-stimulation syndrome (OHSS) patients. This suggests that ovary dysfunction may lead to ANGII accumulation in ascites [63]. We have confirmed that ANGII in ascites from ovarian cancer patients is significantly higher than from non-cancerous patients. The basal level of ANGII in peritoneal fluid is extremely low. Hence, there is potential to use ANGII as a biomarker for the diagnosis of ovarian cancer recurrence in the peritoneal cavity. However, more extensive studies with larger patient numbers are required to confirm this hypothesis.

## Conclusions

In this study, the molecular mechanism by which AGTR1 contributes to ovarian cancer has been identified. A summary is depicted in Fig. 6g. The results of this study provide a foundational understanding of the importance of AGTR1 in MCS formation and also explains the association among high levels of AGTR1 and poor clinical outcomes in EOC patients. For the first times, this study demonstrates a relationship between the ANGII/AGTR1 signaling axis and desaturation of fatty acid biogenesis in EOC spheroid formation. AGTR1-targeted therapy may be a potential strategy for elimination of EOC peritoneal metastasis.

## Additional files

Additional file 1 **Table S1.** The primers sequences used in this study for RT-qPCR, RT-qPCR: Quantitative reverse transcription PCR. Table S2. The antibodies information and dilution ratio used in this study, CST: Cell Signaling Technology, WB: Western blotting, IHC: Immunohistochemistry. (PDF 185 kb)

Additional file 2 **Figure S1**.| ANGII effect on ovarian cancer cell viability**.** (**a**) The ANGII effect on proliferation of Ovca429 cell were measured by MTT assay. The data is presented as means ± SEM and the significant difference were indicated (*p < 0.05 against control). (**b**) The ANGII effect on proliferation of HM cell were measured by MTT assay. The data is presented as means ± SEM and the significant difference were indicated (*p < 0.05 against control). (**c**) The ANGII effect on proliferation of A2780 cell were measured by MTT assay. The data is presented as means ± SEM and the significant difference were indicated (*p < 0.05,**p < 0.01,***p < 0.001 against control). Figure S2| ANGII promotes ovarian cancer MCS formation and migration. (**a**) ANGII significantly increased the maximum diameter of the MCS. The diameters of the spheroids (at least 10 spheroids counted) in the Matrigel were measured by ImageJ software. (**b**) The cell growth of the ovarian cancer spheroids was measured by crystal violet staining. The growth areas were quantified by ImageJ software. (**c**) The western blot band intensity was determined by the gel imaging system (ChemiDoc™ XRS+ Imaging Systems, Bio-Rad) and data are shown as means ± SEM; * *p* < 0.05, ** *p* < 0.01. (**d**) Bright field images of the cell morphology of the parental cells and migrated cells after the Transwell assay. Scale bar, 100 μm. (**e**) Total RNA were extracted from the parental cells and the migrated cells. The expression of AGTR1 and AGT were determined by RT-qPCR. The relative expression levels of AGTR1 and AGT were calculated by the -2ddCt method. The data are presented as means ± SEM. Significant differences between parental and migrated cells are indicated (* *p* < 0.05, *** *p* < 0.001). Figure S3.| AGTR1 gene expression in ovarian cancer cell line**. (a)** AGTR1 gene relative expression level in A2780, HM and Ovca429 cell were quantified by RT-qPCR. The result is presented as means ± SEM. (**b**) The silencing efficiency of siRNA-AGTR1 on suppressing of AGTR1 mRNA expression level. The result is presented as means ± SEM and the significant difference were indicated (*p < 0.05,***p < 0.001 against NT-siRNA). (**c**) The silencing efficiency of siRNA-AGTR1 was confirmed by Western blotting. (**d**) Three receptor AGTR1, AGTR2 and MAS1 expression level in Ovca429 cell were quantified by RT-qPCR. The result is presented as means ± SEM. Figure S4.| AGTR1 gene expression predicates high metastasis of ovarian cancer cell. (**a**) AGTR1 upregulated in metastatic subtype of ovarian cancer patients. (**b**) The AGTR1 gene expression is significantly positively correlated with EMT markers gene expression (spearman correlation test, *p*-value =3.39e-75). (**c**) GSEA enrichment analysis show the EMT gene set were activated in AGTR1 high expression patients (NES = 1.77, NOM *p* = 0.032, FDR = 0.115). Abbreviation: Epi-A, epithelial-A; Epi-B, epithelial-B; Mes, mesenchymal; Stem-A, stem-like-A; Stem-B, stem-like-B. Figure S5| ANGII triggered classical AGTR1 signaling and the transactivation of EGFR in ovarian cancer cells. **(a)** p-AKT and p-ERK protein level in ovarian cancer cell after ANGII treatment were measured by Western blot and normalized using GAPDH as a loading control. (**b**) p-AKT and p-ERK protein level in ovarian cancer cell under ANGII with/without losartan treatment were measured by Western blot and normalized using GAPDH as a control. (**c**) MMP2, EGFR, p-EGFR protein level in ovarian cancer cell under ANGII treatment were measured by Western blot and normalized using GAPDH as a loading control. (**d**) p-EGFR, p-Gab1 and p-Shc protein level in ovarian cancer under ANGII with/without losartan treatment were measured by Western blot and normalized using GAPDH as a loading control All data are presented as means ± SEM from at least three experiments; * *p* < 0.05, ** *p* < 0.01, *** *p* < 0.001 against the no treatment control or the samples with ANGII treatment. Figure S6| AGTR1 high expression predicates transactivation of EGFR signaling pathway. (**a**) Volcano plot show the proteins upregulated/ downregulated in AGTR1 high expression patients tumor tissues compared with AGTR1 low expression patients tumor tissues. (**b**) The proteins upregulated were analyzed using GO enrichment analysis. Figure S7| ANGII enhances the MCS formation by reducing the cell necrosis **(a)** Cell death of MCS was assessed by Annexin V-FITC and PI assay by flow cytometry after treatment with ANGII (100 nM) and/or losartan (10 μM). Necrotic cells in each group were quantified. The data are presented as means ± SEM from at least three experiments; * *p* < 0.05, *** *p* < 0.001 against the control group. **(b)** Cell death inside MCS were detected by flow cytometry with different combinations of treatment: ANGII (100 nM), losartan (10 μM), CGP42112 (50 nM) and/or ANG(1–7) (100 nM). Necrotic cells in each group were quantified accordingly. The data are presented as means ± SEM from at least three experiments; * *p* < 0.05, ** *p* < 0.01, *** *p* < 0.001 against control. Figure S8| ANGII induced SCD1 expression by upregulation of transcriptional factor SREBP1. **(a & b)** SCD1 and EHHADH protein level in ovarian cancer cell after ANGII treatment were measured by Western blot and normalized using beta-actin as a loading control. (**c** & **d**) SREBP1 and SCD1 protein level in HM cell and Ovca429 cell under ANGII were measured by Western blot and normalized using beta-actin as a control. (**e**) p-ERK1/2, p-AKT protein level in ovarian cancer cell under ERK1/2 inhibitor (PD98059,50 μM) and PI3K/AKT inhibitor (LY294002, 100 μM) treatment were measured by Western blot and normalized using beta-actin as a loading control. All data are presented as means ± SEM from at least three experiments; * *p* < 0.05, ** *p* < 0.01, *** *p* < 0.001 against the no treatment control or the samples with ANGII treatment. Figure S9| Blocking of SCD1 reverse ANGII effect on relieve cancer cell death during spheroid formation. **(a & b)** SCD1 inhibitor (cat: ab142089, 10 nM) reversed the effect of ANGII on necrosis in spheroids of two ovarian cancer cell lines (a: Ovca429 cells, b: HM cells). The necrotic cells were measured by Annexin V-FITC/PI flow cytometry. The data are presented as means ± SEM from at least three experiments; * *p* < 0.05, *** *p* < 0.001 against the control group. (**c**) The knock-down efficiency of shRNA on SCD1 expression was confirmed by Western blotting. (**d**) Knock-down of SCD1 significantly reduced the ANGII mediated spheroid formation of ovarian cancer cell. (PDF 798 kb)

## Abbreviations

ACE: Angiotensin-converting enzyme
AGT: Angiotensinogen
AGTR1: Angiotensin II type 1 receptor
ANGII: Angiotensin II
BiP: Binding immunoglobulin protein
CAM: Construction of constitutive active mutant
EGFR: Epidermal growth factor receptor
EMT: Epithelial-to-mesenchymal transition
EOC: Epithelial ovarian cancer
ER: Endoplasmic reticulum
IHC: Immunohistochemistry
MAS1: Mas-related G-protein coupled receptor
MCS: Multicellular spheroid
MMP2: Metalloproteinase-2
SCD1: Stearoyl-CoA Desaturase-1
SREBP: Sterol regulatory element-binding protein
