# Supplementary material for: Angiotensin II promotes ovarian cancer spheroid formation and metastasis by upregulation of lipid desaturation and suppression of endoplasmic reticulum stress
Source: J Exp Clin Cancer Res. 2019 Mar 7;38:116. doi: 10.1186/s13046-019-1127-x (PMC6407256; doi:10.1186/s13046-019-1127-x)
Supplement: Supplementary file 1 — Table S1. The primers sequences used in this study for RT-qPCR, RT-qPCR: Quantitative reverse transcription PCR. Table S2. The antibodies information and dilution ratio used in this study, CST: Cell Signaling Technology, WB: Western blotting, IHC: Immunohistochemistry. (PDF 185 kb) [file 13046_2019_1127_MOESM1_ESM.pdf]

Table 1. The primers sequences used in this study for RT-qPCR

Gene symbol	Forward primer	Reverse primer
AGTR1	CCTCAGATAATGTAAGCTCATCCAC	GCTGCAGAGGAATGTTCTCTT
AGTR2	AGGCACTAAGCAAGCTGATTT	GCTAGTAGTGGCAAGGGTGG
MAS1	GACCAATGCCGACTGGTACT	ACAACACGGGCCTCTATCTG
S14	GGCAGACCGAGATGAATCCTCA	CAGGTCCAGGGGTCTTGGTCC

RT-qPCR: Quantitative reverse transcription PCR

Table 2. The antibodies information and dilution ratio used in this study

Antibody	Vendor and catalog number	Dilution
EGFR	CST, cat:4267	1:1000 WB
p-EGFR	CST, cat:3777	1:1000 WB
MMP2	CST, cat:40994	1:1000 WB
p-AKT	CST, cat:4060	1:1000 WB
p-ERK	CST, cat:4370	1:1000 WB
p-GAB1	CST, cat:3233	1:1000 WB
p-shc	CST, cat:2434	1:1000 WB
SCD1	CST, cat:2794	1:1000 WB
EHHADH	Santa cruz, cat:sc-393123	1:1000 WB
BiP	CST, cat:3177	1:1000 WB
AGTR1	MyBiosource,cat:MBS244122	1:200 IHC,WB
SREBP1	Thermofisher,cat:ma5-11685	1:500 WB
CHOP	CST, cat:5554T	1:1000 WB
PERK	CST,cat:5683	1:1000 WB
p-PERK	CST, cat:3179S	1:1000 WB
GAPDH	CST, cat:5174	1:1000 WB
Beta-actin	CST, cat:4970	1:1000 WB

CST: Cell Signaling Technology
WB: Western blotting
IHC: Immunohistochemistry
