# Supplementary material for: Angiotensin II promotes ovarian cancer spheroid formation and metastasis by upregulation of lipid desaturation and suppression of endoplasmic reticulum stress
Source: J Exp Clin Cancer Res. 2019 Mar 7;38:116. doi: 10.1186/s13046-019-1127-x (PMC6407256; doi:10.1186/s13046-019-1127-x)
Supplement: Supplementary file 2 — Figure S1.| ANGII effect on ovarian cancer cell viability. (a) The ANGII effect on proliferation of Ovca429 cell were measured by MTT assay. The data is presented as means ± SEM and the significant difference were indicated (*p < 0.05 against control). (b) The ANGII effect on proliferation of HM cell were measured by MTT assay. The data is presented as means ± SEM and the significant difference were indicated (*p < 0.05 against control). (c) The ANGII effect on proliferation of A2780 cell were measured by MTT assay. The data is presented as means ± SEM and the significant difference were indicated (*p < 0.05,**p < 0.01,***p < 0.001 against control). Figure S2| ANGII promotes ovarian cancer MCS formation and migration. (a) ANGII significantly increased the maximum diameter of the MCS. The diameters of the spheroids (at least 10 spheroids counted) in the Matrigel were measured by ImageJ software. (b) The cell growth of the ovarian cancer spheroids was measured by crystal violet staining. The growth areas were quantified by ImageJ software. (c) The western blot band intensity was determined by the gel imaging system (ChemiDoc™ XRS+ Imaging Systems, Bio-Rad) and data are shown as means ± SEM; * p < 0.05, ** p < 0.01. (d) Bright field images of the cell morphology of the parental cells and migrated cells after the Transwell assay. Scale bar, 100 μm. (e) Total RNA were extracted from the parental cells and the migrated cells. The expression of AGTR1 and AGT were determined by RT-qPCR. The relative expression levels of AGTR1 and AGT were calculated by the -2ddCt method. The data are presented as means ± SEM. Significant differences between parental and migrated cells are indicated (* p < 0.05, *** p < 0.001). Figure S3.| AGTR1 gene expression in ovarian cancer cell line. (a) AGTR1 gene relative expression level in A2780, HM and Ovca429 cell were quantified by RT-qPCR. The result is presented as means ± SEM. (b) The silencing efficiency of siRNA-AGTR1 on suppressing of AGTR [file 13046_2019_1127_MOESM2_ESM.pdf]

Supplementary Figure 1

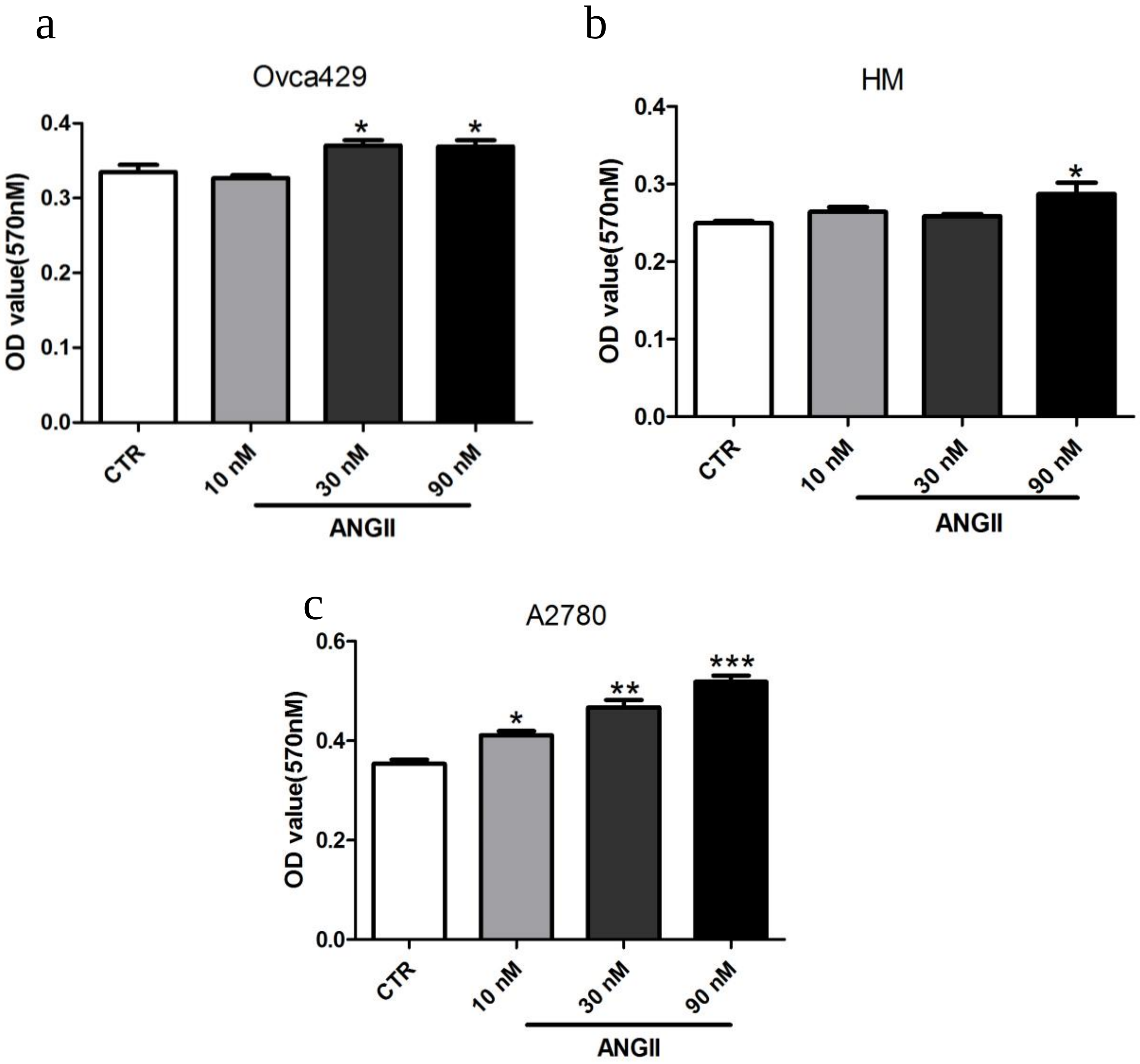

Supplementary Figure 1| ANGII effect on ovarian cancer cell viability. (**a**) The ANGII effect on proliferation of Ovca429 cell were measured by MTT assay. The data is presented as means ± SEM and the significant difference were indicated (*p<0.05 against control). (**b**) The ANGII effect on proliferation of HM cell were measured by MTT assay. The data is presented as means ± SEM and the significant difference were indicated (*p<0.05 against control). (**c**) The ANGII effect on proliferation of A2780 cell were measured by MTT assay. The data is presented as means ± SEM and the significant difference were indicated (*p<0.05,**p<0.01,***p<0.001 against control).

Supplementary Figure 2

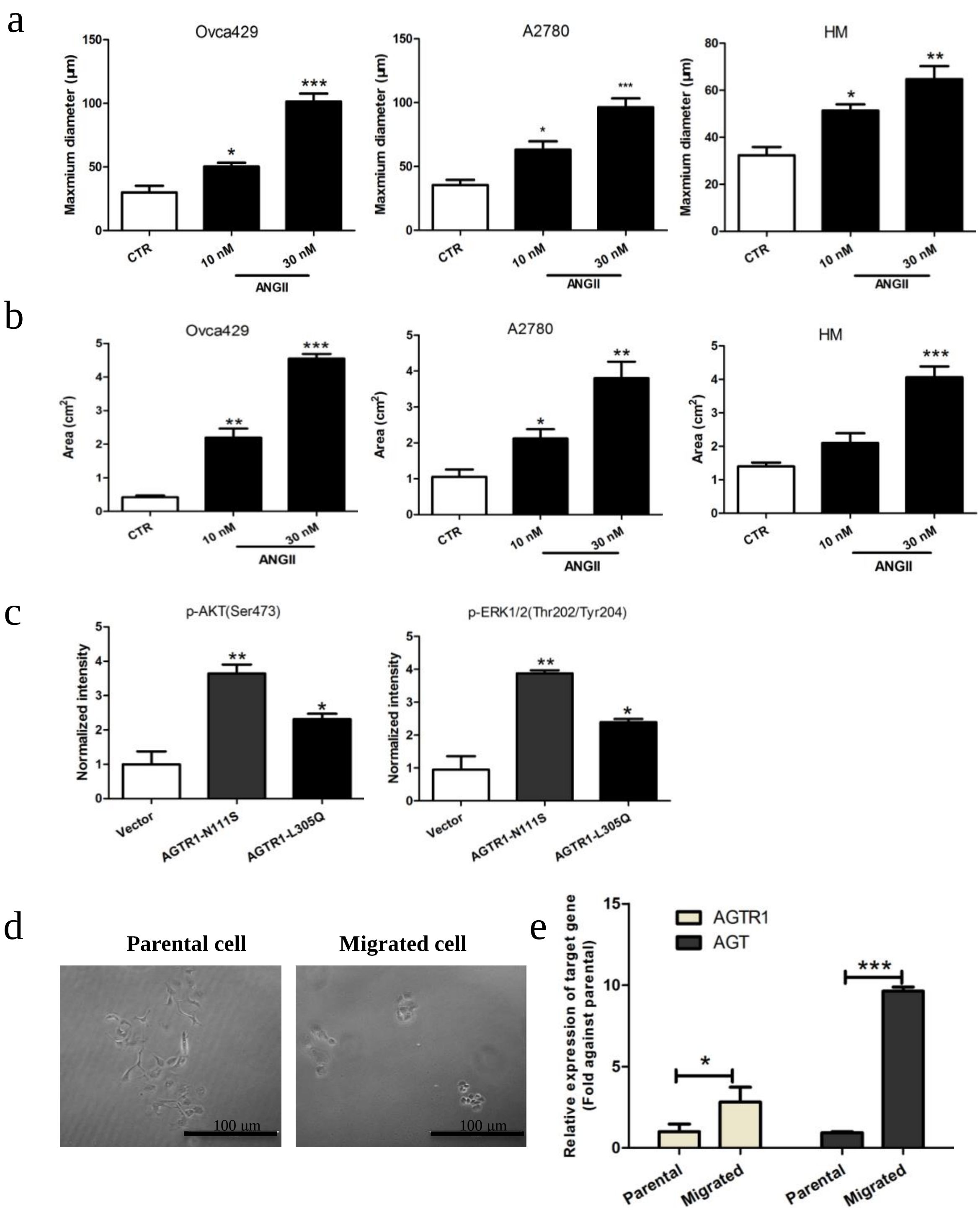

Supplementary Figure 2| ANGII promotes ovarian cancer MCS formation and migration. (**a**) ANGII significantly increased the maximum diameter of the MCS. The diameters of the spheroids (at least 10 spheroids counted) in the Matrigel were measured by ImageJ software. (**b**) The cell growth of the ovarian cancer spheroids was measured by crystal violet staining. The growth areas were quantified by ImageJ software. (**c**) The western blot band intensity was determined by the gel imaging system (ChemiDoc™ XRS+ Imaging Systems, Bio-Rad) and data are shown as means ± SEM; * $p < 0.05$, ** $p < 0.01$. (**d**) Bright field images of the cell morphology of the parental cells and migrated cells after the Transwell assay. Scale bar, 100 µm. (**e**) Total RNA were extracted from the parental cells and the migrated cells. The expression of AGTR1 and AGT were determined by RT-qPCR. The relative expression levels of AGTR1 and AGT were calculated by the -2ddCt method. The data are presented as means ± SEM. Significant differences between parental and migrated cells are indicated (* $p < 0.05$, *** $p < 0.001$).

Supplementary Figure 3

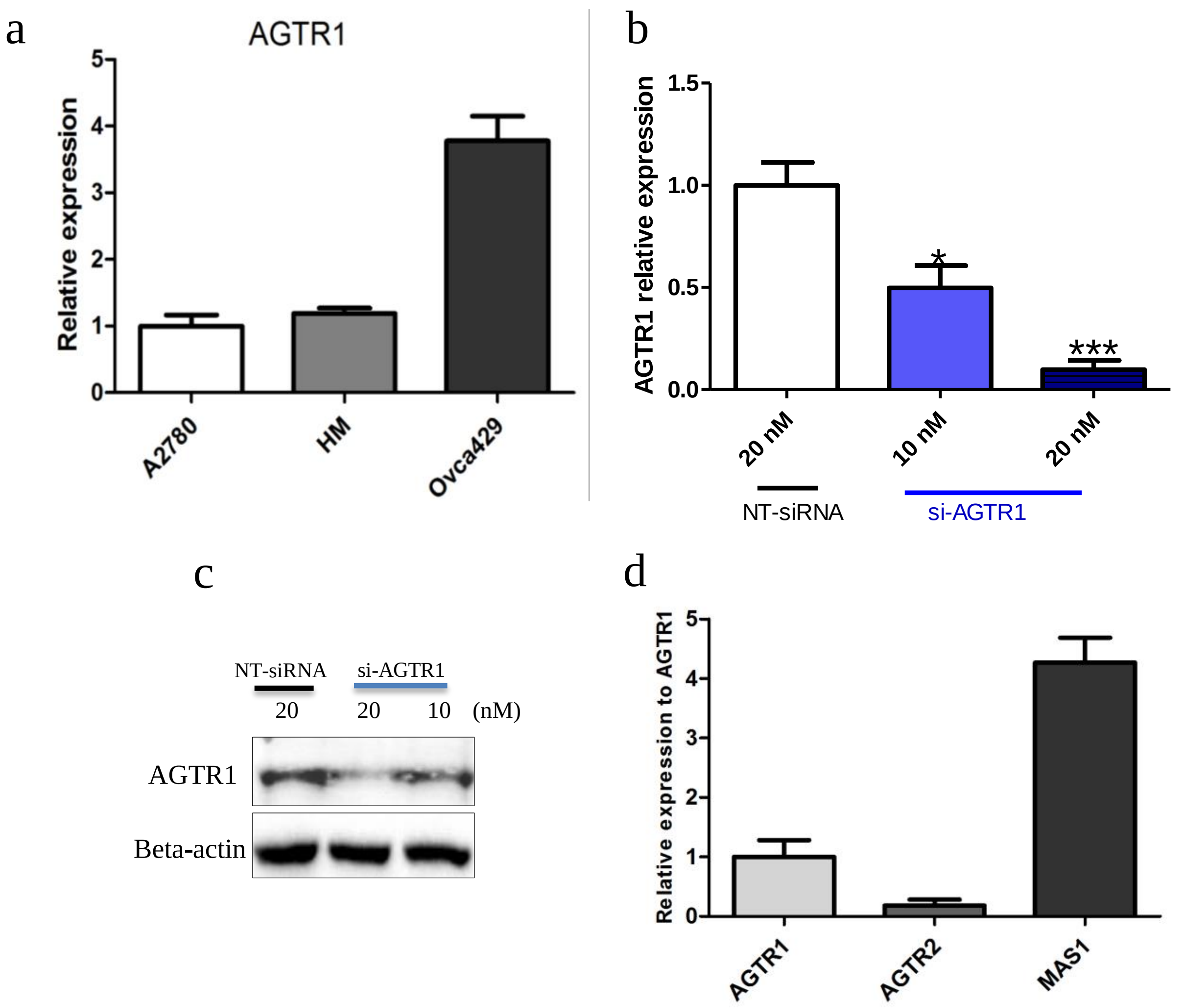

Supplementary Figure 3| AGTR1 gene expression in ovarian cancer cell line. (**a**) AGTR1 gene relative expression level in A2780, HM and Ovca429 cell were quantified by RT-qPCR. The result is presented as means ± SEM. (**b**) The silencing efficiency of siRNA-AGTR1 on suppressing of AGTR1 mRNA expression level. The result is presented as means ± SEM and the significant difference were indicated (*$p<0.05$,***$p<0.001$ against NT-siRNA). (**c**) The silencing efficiency of siRNA-AGTR1 was confirmed by Western blotting. (**d**) Three receptor AGTR1, AGTR2 and MAS1 expression level in Ovca429 cell were quantified by RT-qPCR. The result is presented as means ± SEM.

Supplementary Figure 4

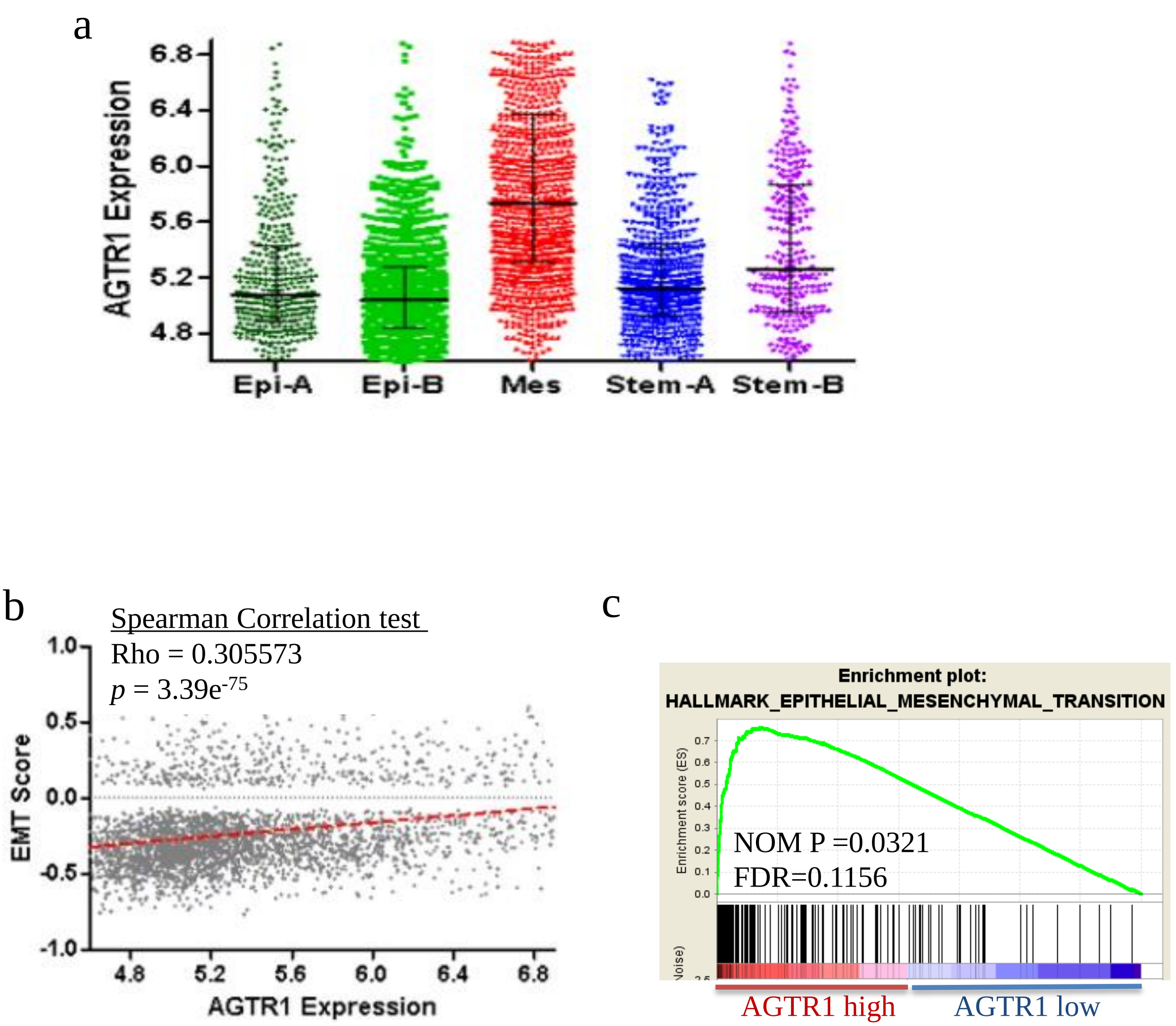

Supplementary Figure 4| AGTR1 gene expression predicates high metastasis of ovarian cancer cell. (**a**) AGTR1 upregulated in metastatic subtype of ovarian cancer patients. (**b**) The AGTR1 gene expression is significantly positively correlated with EMT markers gene expression (spearman correlation test, p-value $=3.39e^{-75}$). (**c**) GSEA enrichment analysis show the EMT gene set were activated in AGTR1 high expression patients (NES=1.77, NOM p=0.032, FDR=0.115). Abbreviation: Epi-A, epithelial-A; Epi-B, epithelial-B; Mes, mesenchymal; Stem-A, stem-like-A; Stem-B, stem-like-B**.**

Supplementary Figure 5

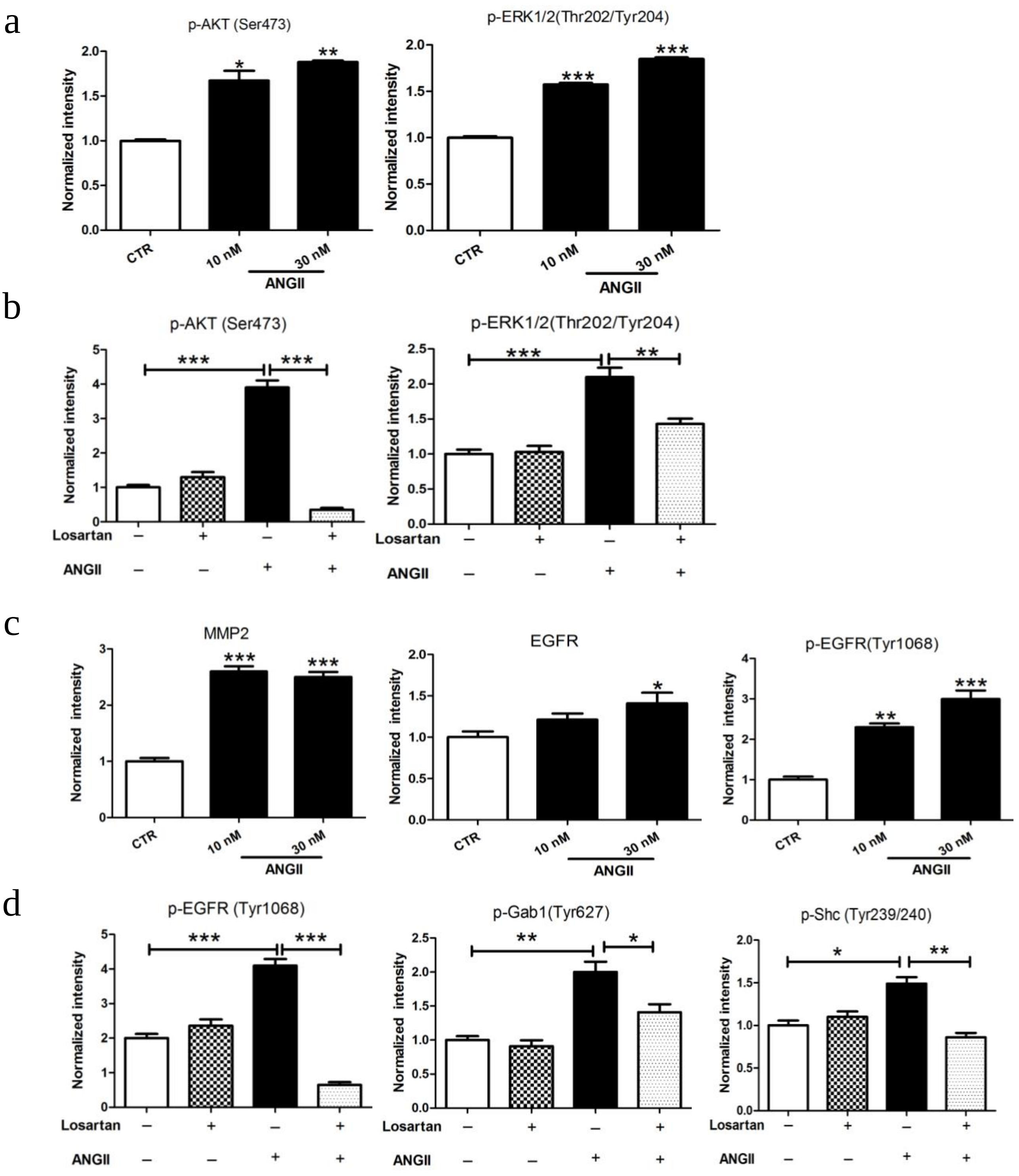

Supplementary Figure 5| ANGII triggered classical AGTR1 signaling and the transactivation of EGFR in ovarian cancer cells. (a) p-AKT and p-ERK protein level in ovarian cancer cell after ANGII treatment were measured by Western blot and normalized using GAPDH as a loading control. (**b**) p-AKT and p-ERK protein level in ovarian cancer cell under ANGII with/without losartan treatment were measured by Western blot and normalized using GAPDH as a control.(**c**) MMP2, EGFR, p-EGFR protein level in ovarian cancer cell under ANGII treatment were measured by Western blot and normalized using GAPDH as a loading control. (**d**) p-EGFR, p-Gab1 and p-Shc protein level in ovarian cancer under ANGII with/without losartan treatment were measured by Western blot and normalized using GAPDH as a loading control All data are presented as means ± SEM from at least three experiments; * $p < 0.05$, ** $p < 0.01$, *** $p < 0.001$ against the no treatment control or the samples with ANGII treatment.

Supplementary Figure 6

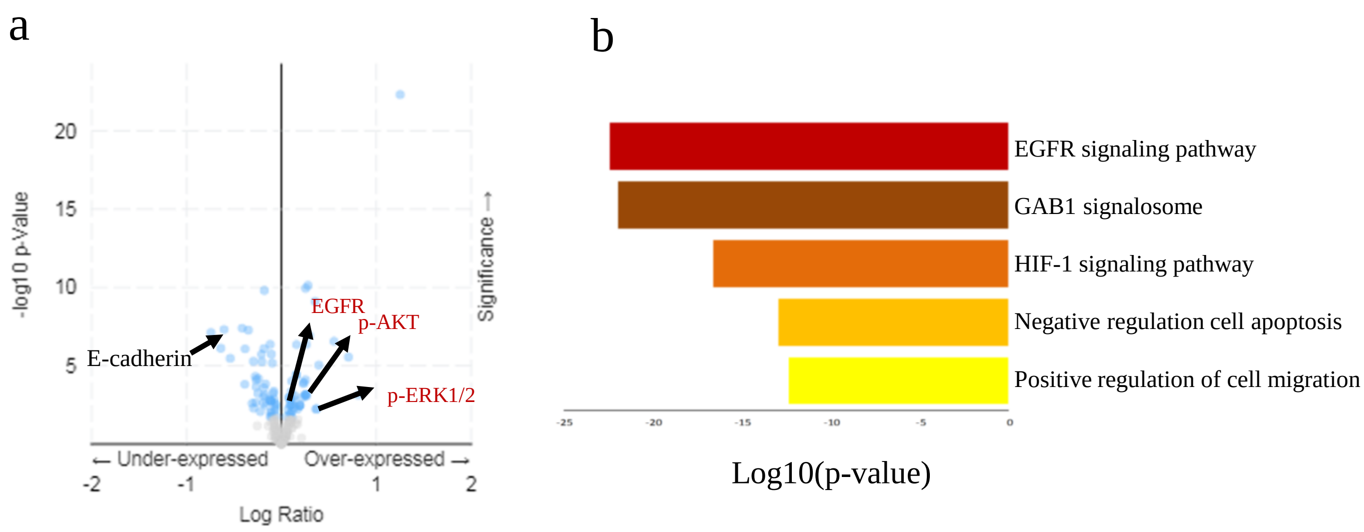

Supplementary Figure 6| AGTR1 high expression predicates transactivation of EGFR signaling pathway. (**a**) Volcano plot show the proteins upregulated/ downregulated in AGTR1 high expression patients tumor tissues compared with AGTR1 low expression patients tumor tissues. (**b**) The proteins upregulated were analyzed using GO enrichment analysis.

Supplementary Figure 7

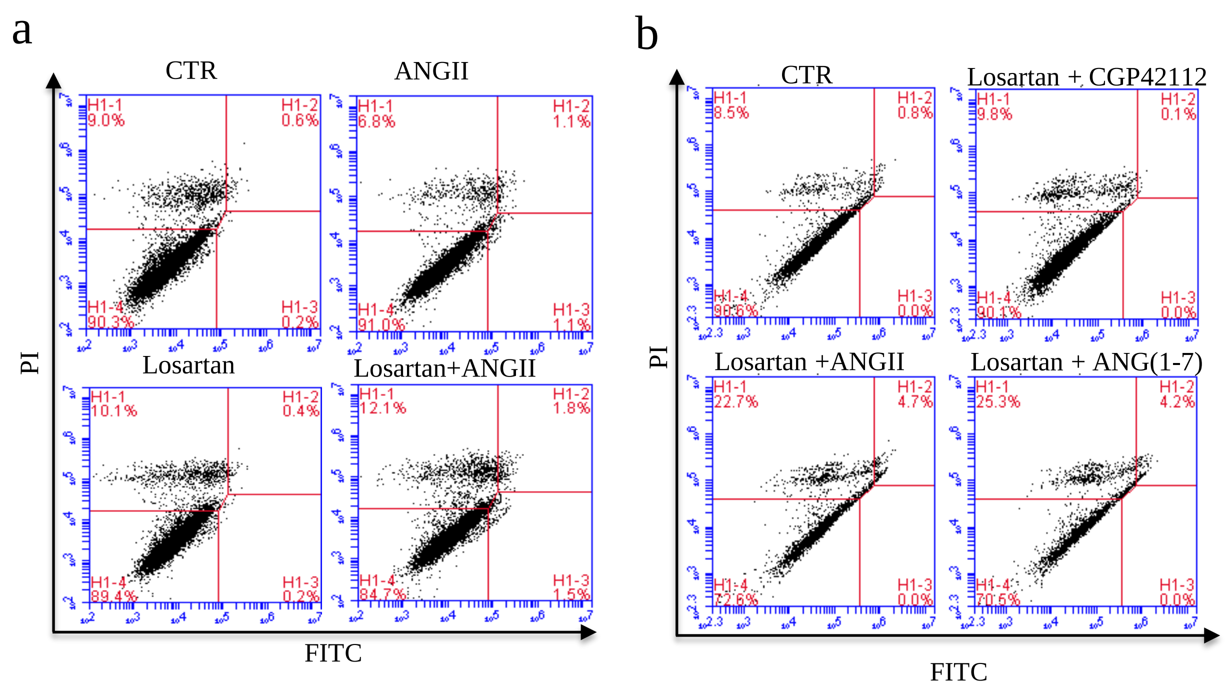

Supplementary Figure 7| ANGII enhances the MCS formation by reducing the cell necrosis (a) Cell death of MCS was assessed by Annexin V-FITC and PI assay by flow cytometry after treatment with ANGII (100 nM) and/or losartan (10 μM). Necrotic cells in each group were quantified. The data are presented as means ± SEM from at least three experiments; * $p < 0.05$, *** $p < 0.001$ against the control group. **(b)** Cell death inside MCS were detected by flow cytometry with different combinations of treatment: ANGII (100 nM), losartan (10 μM), CGP42112 (50 nM) and/or ANG(1-7) (100 nM). Necrotic cells in each group were quantified accordingly. The data are presented as means ± SEM from at least three experiments; * $p < 0.05$, ** $p < 0.01$, *** $p < 0.001$ against control.

Supplementary Figure 8

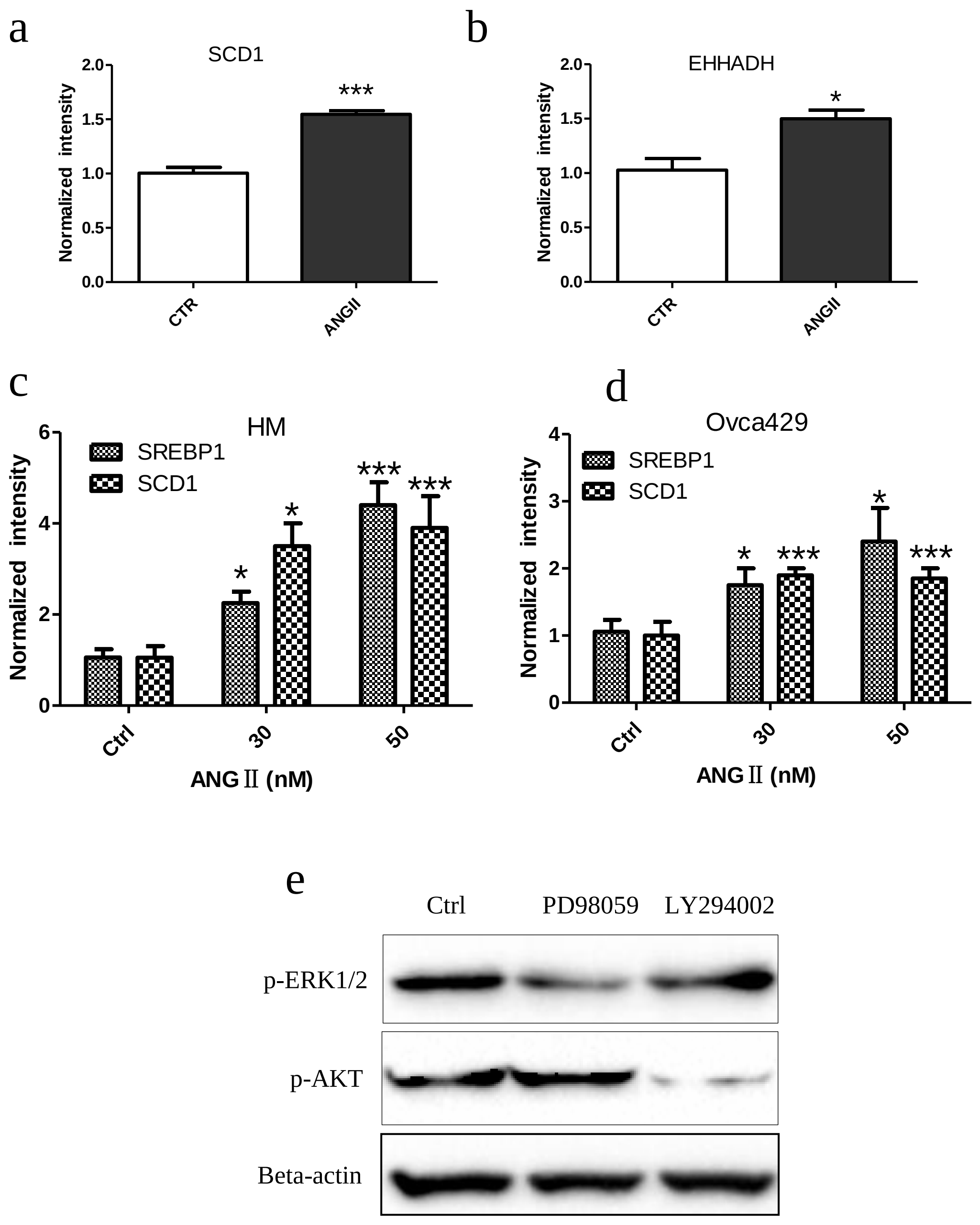

Supplementary Figure 8| ANGII induced SCD1 expression by upregulation of transcriptional factor SREBP1. (a & b) SCD1 and EHHADH protein level in ovarian cancer cell after ANGII treatment were measured by Western blot and normalized using beta-actin as a loading control. (**c** & **d**) SREBP1 and SCD1 protein level in HM cell and Ovca429 cell under ANGII were measured by Western blot and normalized using beta-actin as a control. (**e**) p-ERK1/2, p-AKT protein level in ovarian cancer cell under ERK1/2 inhibitor(PD98059,50μM) and PI3K/AKT inhibitor(LY294002, 100μM) treatment were measured by Western blot and normalized using beta-actin as a loading control. All data are presented as means ± SEM from at least three experiments; * $p < 0.05$, ** $p < 0.01$, *** $p < 0.001$ against the no treatment control or the samples with ANGII treatment.

Supplementary Figure 9

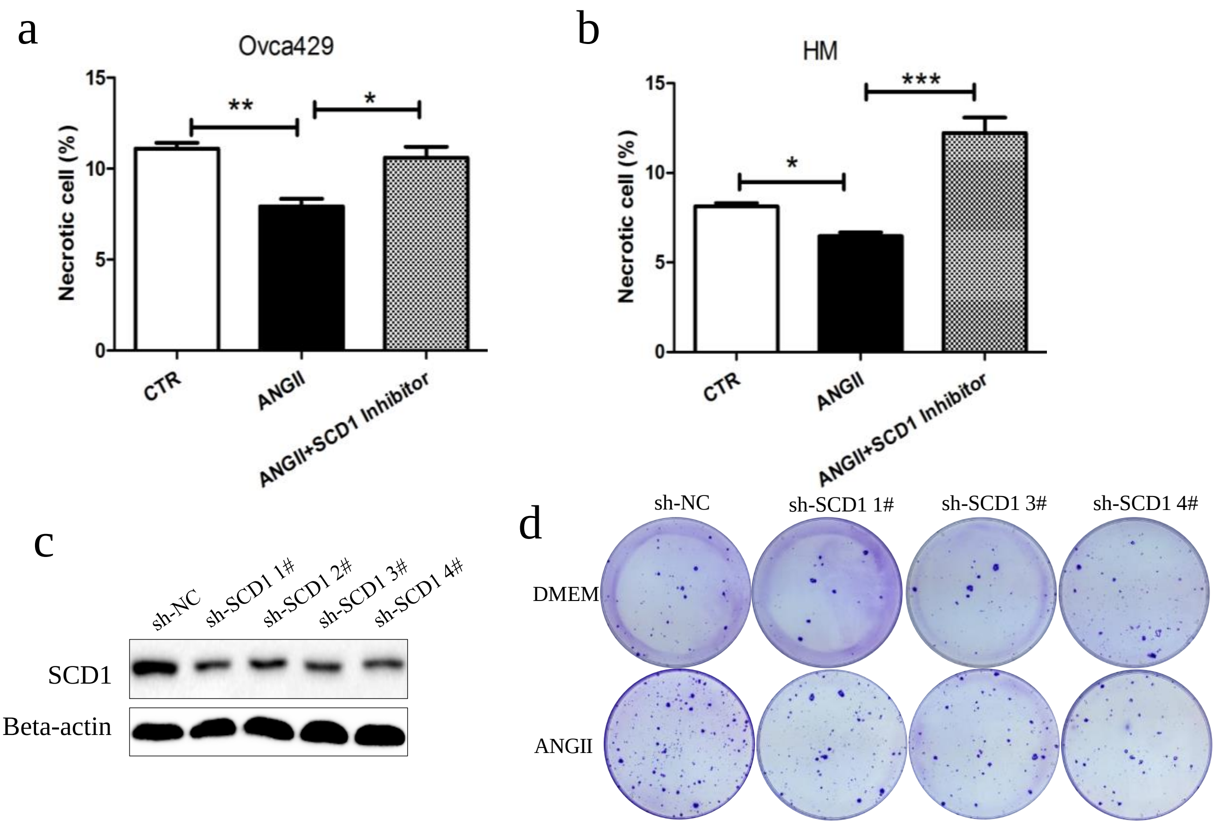

Supplementary Figure 9| Blocking of SCD1 reverse ANGII effect on relieve cancer cell death during spheroid formation. (a & b) SCD1 inhibitor (cat: ab142089, 10 nM) reversed the effect of ANGII on necrosis in spheroids of two ovarian cancer cell lines (a: Ovca429 cells, b: HM cells). The necrotic cells were measured by Annexin V-FITC/PI flow cytometry. The data are presented as means ± SEM from at least three experiments; * $p < 0.05$, *** $p < 0.001$ against the control group. (**c**) The knock-down efficiency of shRNA on SCD1 expression was confirmed by Western blotting. (**d**) Knock-down of SCD1 significantly reduced the ANGII mediated spheroid formation of ovarian cancer cell.
